# Enhancing the Photocatalytic Activity of Zirconium‐Based Metal–Organic Frameworks Through the Formation of Mixed‐Valence Centers

**DOI:** 10.1002/advs.202303206

**Published:** 2023-08-07

**Authors:** Zihao Wei, Shaojia Song, Hongfei Gu, Yaqiong Li, Qi Sun, Ning Ding, Hao Tang, Lirong Zheng, Shuhu Liu, Zhenxing Li, Wenxing Chen, Shenghua Li, Siping Pang

**Affiliations:** ^1^ School of Materials Science & Engineering Beijing Institute of Technology Beijing 100081 P. R. China; ^2^ State Key Laboratory of Heavy Oil Processing China University of Petroleum Beijing 102249 China; ^3^ Institute of High Energy Physics Chinese Academy of Science Beijing 100049 China; ^4^ Energy & Catalysis Center School of Materials Science and Engineering Beijing Institute of Technology Beijing 100081 China

**Keywords:** cellulose acetate, formate, interface effect, metal–organic framework, photocatalytic CO_2_ reduction

## Abstract

Despite the desirability of metal–organic frameworks (MOFs) as heterogeneous photocatalysts, current strategies available to enhance the performance of MOF photocatalysts are complicated and expensive. Herein, a simple strategy is presented for improving the activity of MOF photocatalysts by regulating the atomic interface structure of the metal active sites on the MOF. In this study, MOF (PCN‐222) is hybridized with cellulose acetate (CA@PCN‐222) through an optimized atomic interface strategy, which lowers the average valence state of Zr ions. The electronic metal‐support interaction mechanism of CA@PCN‐222 is revealed by evaluating the photocatalytic CO_2_ reduction reaction (CO_2_RR). The experimental results suggested that the electron migration efficiency at the atomic interface of the MOFs strongly coupled with cellulose is significantly improved. In particular, the CO_2_RR to formate activity of CA@PCN‐222 photocatalyst greatly increased from 778.2 to 2816.0 µmol g^−1^ compared with pristine PCN‐222 without cellulose acetate. The findings suggest that the strongly coupled metal–ligand moiety at the atomic interface of MOFs may play a synergistic role in heterogeneous catalysts.

## Graphical abstract

MOF (PCN‐222) is hybridized with cellulose acetate (CA@PCN‐222) through an optimized atomic interface strategy, which lowers the average valence state of Zr ions. The CA@PCN222 exhibits high photocatalytic activity of CO_2_RR.

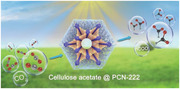

## 1 Introduction

Metal−organic frameworks (MOFs) are porous materials with large surface areas formed by metal ions or clusters connected by multiple organic linkers.^[^ ^1^ ^]^ As an emerging subclass, zirconium‐based MOFs (Zr‐MOFs; e.g., PCN‐222, UiO‐66, NH_2_‐UiO‐66, and NU‐1000)^[^ ^2^ ^]^ have drawn considerable interest as heterogeneous photocatalysis for potential applications, such as H_2_O splitting,^[^ ^3^ ^]^ CO_2_ reduction,^[^ ^4^ ^]^ H_2_ production,^[^ ^5^ ^]^ and dye degradation,^[^ ^6^ ^]^ because these MOFs possess extremely high thermal and chemical stabilities owing to strong coordination bonds between their Zr clusters and organic linkers and can work under complicated and harsh catalytic conditions. Moreover, Zr ions have variable redox states (e.g., Zr^4+^/Zr^3+^) and their clusters behave as inorganic semiconductor quantum entities, and organic linkers, such as porphyrin and salen, can serve as antennae to activate these metal clusters upon photoexcitation.^[^ ^7^ ^]^ Despite these advantages, their photocatalytic activities are not satisfactory, primarily because the energy bands between the highest occupied molecular orbital (HOMO) and least unoccupied molecular orbital (LUMO) are relatively wide, making photogenerated electron transfer from the organic linkers to the Zr clusters difficult.

**Figure 1:**
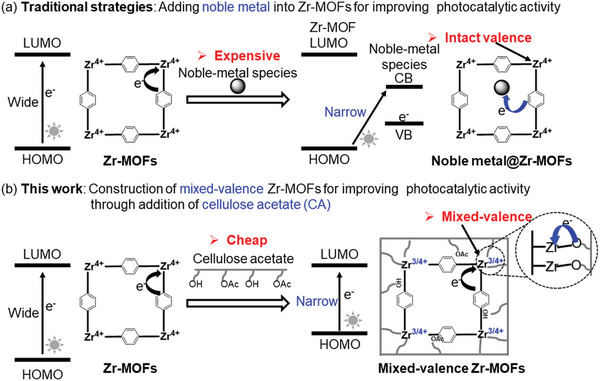
Strategies for enhancing the photocatalytic activity of MOF‐based photocatalysts. a) Traditional, expensive, and stable strategy. b) Cheap and stable in situ strategy using cellulose to adjust the structure of the MOF's atomic interface (this study).

To enhance the photocatalytic performance of Zr‐MOFs, one of the most commonly used strategies is the introduction of noble metal (e.g., Pd, Au, and Ir; **Figure** **1**a)^[^ ^8^ ^]^ species, including nanoparticles and nanoclusters, into their pores to form photoactive composites. Because noble metal species usually have extremely narrow energy bands, introducing these materials can endow the resultant composites with narrower energy bands between the HOMO of Zr‐MOFs and the CB of noble metal species compared with pristine MOFs, which facilitates the transfer of photogenerated electrons from the organic ligands to the noble metal species. The introduction of noble metals can provide more active sites. Many Zr‐MOF composites, such as Au@UiO‐66,^[^ ^9^ ^]^ Pd@UiO‐66,^[^ ^10^ ^]^ and Pt@PCN‐222^[^ ^11^ ^]^ have been developed, which exhibit remarkably higher photocatalytic activities for nitrogen fixation and CO_2_ reduction than pristine MOFs. However, the preparation method of most of these composites is complicated and requires expensive noble metals. Thus, the development of a simple and inexpensive strategy for enhancing the photocatalytic activity of Zr‐MOFs is highly desirable.

However, an interfacial engineering strategy^[^ ^12^ ^]^ has been originally developed to improve the photocatalytic performances of photoactive MOFs by adjusting the electron distribution of the metal active site, such as ligands, additives, solvents used, and temperatures. In these interfacially engineered MOFs, the optimized electronic structure of the metal ions can make the energy bands narrower than those of pristine MOFs, which facilitates the transfer of photogenerated electrons from organic ligands to metal centers, thus improving their photocatalytic activities. For example, Zhan et al.^[^ ^13^ ^]^ employed this strategy to create novel heterostructures by coating rutile TiO_2_ nanorods with UiO‐67 and enhancing the internal electric field (IEF) at the heterojunction interface, resulting in higher photocatalytic water oxidation compared with pristine TiO_2_. Interfacial engineering can be rationally designed for MOF catalysis based on unique physicochemical properties to improve the catalytic activity and selectivity. The interactions between MOFs and reactants are critical for catalytic reactions and depend largely on the atomic arrangement and electronic structure of the interface.^[^ ^14^ ^]^ Therefore, to obtain better MOF catalytic performance, it is necessary to adjust the electron distribution of the MOFs using a reasonable structural design.

As a natural polymer, cellulose acetate (CA)^[^ ^15^ ^]^ is readily available, inexpensive, and eco‐friendly. CA contains abundant electron‐donating oxygen‐containing groups, such as hydroxyl and acetyl groups, and possesses strong coordination abilities. Based on the attractive features of CA, in this study, we proposed an effective strategy (Figure 1b) for improving the photocatalytic performance of Zr‐MOFs by introducing CA into Zr‐MOFs and forming interface‐optimized Zr‐MOF composites via a simple one‐pot reaction. In the resultant composites, strong coordination interactions were formed between the oxygen‐containing groups of cellulose and Zr^4+^ ions of the Zr‐MOFs. Owing to their strong electron‐donating abilities, the oxygen atoms of the coordinated groups can transfer their electrons to their adjacent Zr^4+^ ions at the interface of these composites, resulting in a lower valence state (Zr^3+^) and lower energy bands, and facilitating photogenerated electron transfer. We anticipated that the interface‐optimized Zr‐MOF composites would exhibit higher photocatalytic activities than pristine Zr‐MOFs. Thus, compared with the introduction of traditional noble metals, the proposed strategy is simpler and cheaper.

To examine the viability of our strategy, PCN‐222^[^ ^16^ ^]^ was employed as a typical example because it possesses strong visible‐light adsorption ability owing to the incorporation of a porphyrin ligand. The structure and photocatalytic activity of PCN‐222 for CO_2_ reduction are well established, facilitating comparison. To further enhance its photocatalytic activity, CA was introduced into PCN‐222 to form an interface‐optimized PCN‐222 composite (CA@PCN‐222). The structure of this composite was characterized using various methods, such as Powder X‐ray diffraction pattern (PXRD), X‐ray photoelectron spectra, and hard X‐ray absorption fine structure (XAFS). Their photoelectrochemical properties and photocatalytic performances in CO_2_ reduction were explored in detail.

## 2 Results and Discussion

### 2.1 Synthesis and Morphology Characterization

**Figure 2:**
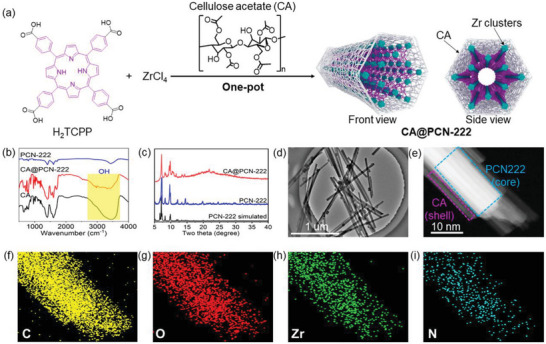
Synthesis and morphology characterizations of CA@PCN‐222. a) Schematic of the synthetic procedures of CA@PCN‐222. b) FT‐IR for CA@PCN‐222 and PCN‐222. c) XRD for CA@PCN‐222 and PCN‐222. d,e) TEM images and f–i) elemental mapping images of CA@PCN‐222.

Following the method described in the previously published studies, PCN‐222 was prepared via a one‐pot reaction between tetrakis ((4‐carboxyphenyl)‐porphyrin (H_2_TTPP)) and ZrCl_4_. Under the same conditions, a mixed‐valence PCN‐222 composite (CA@PCN‐222) was obtained by adding CA to the reaction mixture (**Figure** **2**a). The Fourier transform infrared spectra (FTIR) of CA@PCN‐222 (Figure 2b) showed strong typical absorption peaks at 3300 cm^−1^ associated with the OH groups of CA, indicating that CA was successfully combined with PNC‐222. The Powder X‐ray diffraction patterns of CA@PCN‐222 and pristine PCN‐222 were almost consistent (Figure 2c), indicating that the framework structure of PCN‐222 in CA@PCN‐222 remained intact. The Transmission Electron Microscopy images (Figure 2d,e) of CA@PCN‐222 showed a rod‐shaped morphology consistent with that of PCN‐222. Furthermore, CA@PCN‐222 had a core‐shell structure, with PCN‐222 on the inside and cellulose acetate on the outside. And PCN‐222 had good lattice stripes in Figure S1 (Supporting Information), while CA@PCN‐222 had no lattice stripes in Figure S2 (Supporting Information). The energy‐dispersive X‐ray spectroscopy (EDS) elemental mapping results confirmed that the C, O, Zr, and N species were evenly distributed in CA@PCN‐222 (Figure 2f–i) and PCN‐222 (Figure S4, Supporting Information). And energy spectra of zirconium and carbon on top of each other were superimposed in Figure S3 (Supporting Information). The results better demonstrate the successful synthesis of the core‐shell structure. Inductively coupled plasma optical emission spectroscopy (ICP‐OES) revealed that the Zr (IV) content in CA@PCN‐222 and PCN‐222 was 11.1 and 16.3 wt.%. Elemental analysis showed that the contents of C, H, O, and N elements in CA@PCN‐222 were 46.43%, 3.20%, 21.38%, and 4.36%, respectively, whereas those in PCN‐222 were 50.62%, 4.55%, 18.55%, and 5.37%, respectively. Based on the results of ICP and elemental analysis, the CA content in CA@PCN‐222 was ≈19.87%. Thermal Gravimetric Analyzer techniques (Figures S5 and S6, Supporting Information) indicated that the samples have still maintained the original skeleton at 400 °C, showing high thermal stability.

### 2.2 Chemical State and Atomic Structure Analysis

**Figure 3:**
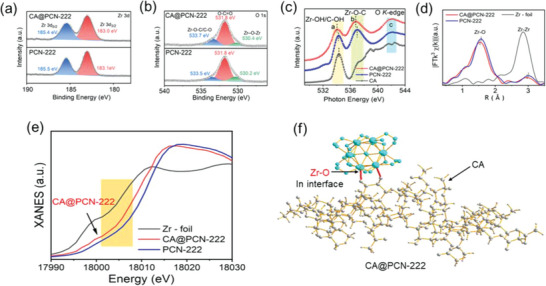
Chemical state and atomic local structure of CA@PCN‐222. High‐resolution XPS spectra of CA@PCN‐222 and pristine PCN‐222: a) Zr 3d region and b) O 1s region. c) O K‐edge XANES spectra of the CA@PCN‐222, pristine PCN222, and cellulose acetate. d) FT k^3^‐weighted Zr K‐edge EXAFS spectra of CA@PCN‐222 and the references. e) C K‐edge XANES spectra of CA@PCN‐222 and pristine PCN‐222. f) Schematic atomic interface model of CA@PCN‐222.

To determine the interfacial circumstance within CA@PCN‐222, X‐ray photoelectron spectra (XPS) were employed for detailed analysis (Figures S6–S9, Supporting Information). As shown in **Figure** **3**a, the binding energies of pristine PCN‐222 for Zr 3d_5/2_ and Zr 3d_3/2_ were 183.1 and 185.5 eV, respectively, and those of CA@PCN‐222 were 183.0 and 185.4 eV, respectively; however, both the peaks shifted negatively by 0.1 eV. Moreover, the peaks of O 1s (Figure 3b) for PCN‐222 located at 533.5 eV (14.7%), 531.8 eV (75.5%), and 530.2 eV (9.9%) were separately originated from the Zr─O─C, O─C═O, and Zr─O─Zr. By contrast, those of CA@PCN‐222 were located at 533.7 eV (11.8%), 531.8 eV (74.3%), and 530.4 eV (13.9%), and the peaks of the Zr─O─C and Zr─O─Zr displayed negative shifts of 0.2 and 0.2 eV, respectively. The content of Zr─O─C greatly decreased from 14.7% (PCN‐222) to 11.8% (CA@PCN‐222), whereas those of O─C═O and Zr─O─Zr correspondingly changed. In addition, XANES spectra showed the same result (Figure 3c; Figure S10, Supporting Information). The above results indicate that the introduction of CA could lead to the formation of strong interactions between CA and PCN‐222 at the interface of CA@PCN‐222, thus resulting in the negative shifts of the Zr and O peaks and a higher content of Zr─O─C compared with those of PCN‐222.

To reveal the interfacial structure and valence state of Zr ions in CA@PCN‐222 at the atomic level, XAFS investigations were performed. Generally, the absorption threshold point of the Zr K‐edge can be used to describe the typical valence state of the Zr ions. In the XANES curves (Figure 3e; Figures S11–S13, Supporting Information), the Zr K–edge position of CA@PCN‐222 was located between the Zr foil (a Zr valence state of 0) and PCN‐222 (a Zr valence state of 4). This clearly indicated that the average valence state of Zr ions was 3.6, which could result from the presence of ≈60% of Zr^4+^ ions and 40% of Zr^3+^ in CA@PCN‐222. As expected, the introduction of CA resulted in a lower average valence state of the Zr ions in CA@PCN‐222 than in PCN‐222.

To further demonstrate why CA@PCN‐222 has a lower average valence state of Zr ions, FT was used to extract and interpret the data of EXAFS spectra. The FT‐EXAFS spectrum of CA@PCN‐222 is shown in Figure 3d. The sample had one clearly visible FT peak at 1.5 Å, which was primarily due to the scattering of Zr─O or Zr─C coordination. Furthermore, the Zr coordination–derived scattering peaks were observed at 3.2 Å. Compared with the PCN‐222 reference, these showed atomic dispersion of Zr clusters and confirmed the presence of a short‐range atomic strain at the interface between cellulose acetate and PCN‐222. The wavelet transform (WT) is regarded as an excellent addition to FT (Figures S14–S17, Supporting Information). Additionally, WT‐EXAFS was used to study the atomic structure of CA@PCN‐222 owing to its powerful resolution in both k and R spaces. Figures S18 and S19 (Supporting Information) show the Zr K–edge WT‐EXAFS spectra of the samples. The Zr─O coordination was shown by one intensity maximum at 2.0 Å^−1^ in the WT contour plots of the CA@PCN‐222. The structural characteristics were extracted using quantitative EXAFS fitting, and the results are shown in Figures S20 and S21 and Table S1 (Supporting Information). Based on EXAFS fitting, Zr was believed to be atomically fixed in the matrix and was sixfold coordinated by O atoms. The Zr─O distance in CA@PCN‐222 appeared to demonstrate an obvious low‐R move from 1.95 to 1.92 Å compared with that in pristine PCN‐222, in addition to the distance between Zr─Zr (from 3.72 to 3.70 Å). This further implied that the local structure of the Zr active sites changed, which was derived from the interaction between CA and PCN‐222. The existence of cluster‐like Zr atoms was confirmed by fusing the morphological data with the associated structural characterizations. This clearly indicates that at the CA@PCN‐222 interface, every Zr atom in PCN‐222 is linked to one oxygen atom of the hydroxyl group in CA through the Zr─O coordination bond (Figure 3f). Thus, because of the strong electron‐donating ability of the oxygen atoms in CA and strong coordination interactions between CA and PCN‐222 in CA@PCN‐222, the coordinated oxygen atom could transfer its electrons to the adjacent Zr ions, resulting in a lower average valence state of the Zr ions in CA@PCN‐222.

### 2.3 Photoelectrochemical Properties

**Figure 4:**
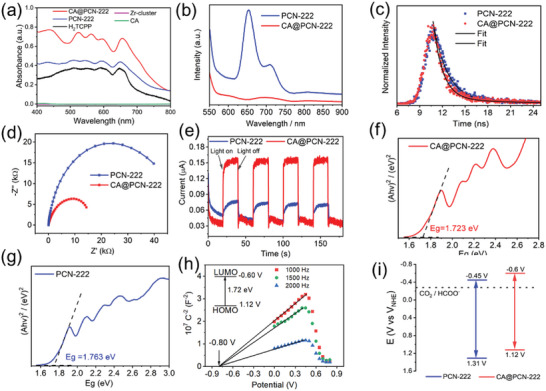
CO_2_ reduction reaction (CO_2_RR) activity of CA@PCN‐222. a) UV/vis spectra of CA@PCN‐222, pristine PCN‐222, H_2_TCPP, Zr‐cluster, and CA. b) Photoluminescence spectra emissions of CA@PCN‐222 and pristine PCN‐222 (at the same concentration in acetonitrile) under excitation at 515 nm. c) Transient fluorescence spectrum of CA@PCN‐222 and pristine PCN‐222. d) Alternating‐current impedance of CA@PCN‐222 and pristine PCN‐222. e) Transient photocurrent responses of CA@PCN‐222 and pristine PCN‐222. f) (A*hf*)^2^ curve of CA@PCN‐222. g) (A*hf*)^2^ curve of PCN‐222. h) Mott–Schottky curve of CA@PCN‐222 at 1000, 1500, and 2000 Hz. i) Diagram of the energy band structure of CA@PCN‐222 and pristine PCN‐222.

The lower average valence state of Zr ions compared with that of PCN‐222 motivated us to investigate the photoactivity of CA@PCN‐222. UV–vis absorption (**Figure** **4**a) showed that CA and Zr‐cluster have no light absorption in the visible region from 400 to 800 nm. However, CA@PCN‐222 has broad and strong adsorption in the region of 400–800 nm owing to the feature of the porphyrin ligand, which is consistent with that of PCN‐222. Under the visible‐light irradiation, the H_2_TCPP ligand will be excited and transfer electron to Zr‐cluster. Thus, the porphyrin ligand plays a role in capturing visible light. Steady‐state photoluminescence (Figure 4b) was measured from 550 to 900 nm under excitation at 515 nm. Notably, the emission intensity of CA@PCN‐222 was lower than that of PCN‐222, and pronounced PL emission quenching was observed. The emission lifetime (Figure 4c) of CA@PCN‐222 was 2.925 ns, which was significantly shorter than that of pristine PCN‐222 (3.176 ns). The obvious PL quenching indicated faster photogenerated electron transfer from the porphyrin ligand to the Zr clusters, and even more efficient electron transfer can be achieved through the introduction of CA. Photocurrent measurements revealed the charge‐separation efficiency of CA@PCN‐222 and agree with the PL results. The intensity of the transient photocurrent of CA@PCN‐222 was significantly higher than that of PCN‐222. The diameter of the semicircular curve of CA@PCN‐222 (Figure 4d) was remarkably smaller than that of PCN‐222, indicating that CA@PCN‐222 has a smaller resistance. Steady‐state surface photovoltage spectra of CA@PCN‐222 and pristine PCN‐222 are measured in Figure S22 (Supporting Information). And the intensity of light response for CA@PCN‐222 is increased from 400 to 800 nm. The significant increase in light absorption activity of CA@PCN‐222 after the addition of cellulose acetate. Moreover, photocurrent measurements (Figure 4e) were performed to explore the characteristics of charge separation and formation caused by visible light, which indicated that the photogenerated charges of CA@PCN‐222 have a better ability to migrate. The electron spin resonance (ESR) spectra (Figure S23, Supporting Information) showed similar results. Thus, these results demonstrated that the introduction of CA greatly suppresses radiative electron‐hole recombination, facilitating photogenerated electron transfer.

To further elucidate the reason for the more efficient photogenerated electron transfer in CA@PCN‐222 than in PCN‐222, their bandgaps were calculated using the Kubelka–Munk transformation. The bandgap of CA@PCN‐222 was 1.72 eV, which was narrowed down by 0.04 eV compared with that of PCN‐222 (1.76 eV; Figure 4f,g). As expected, the lower average valence state of the Zr ions endows CA@PCN‐222 with a narrower bandgap, and the Zr atoms can transition to an excited state more easily.

To understand the potential of CA@PCN‐222 as a photocatalyst, Mott–Schottky experiments were performed at frequencies of 1000, 1500, and 2000 Hz to elucidate the semiconductor characteristics (Figure 4h). The positive slope of the resulting C^−2^ values (vs the applied potentials) matched those of typical n‐type semiconductors. The flat band position derived from the intersection was −0.80 V versus Ag/AgCl (i.e., −0.60 V vs NHE), and the intersection point was frequency independent. It is common knowledge that the flat‐band potential is approximately equal to the bottom of the conduction band (LUMO) in n‐type semiconductors. Moreover, the valence band (HOMO) of CA@PCN‐222 was then calculated to be 1.12 V versus NHE and its LUMO band was −0.6 V versus NHE, which was remarkably lower than that of PCN‐222 (−0.45 V vs NHE; Figure S24, Supporting Information). The more positive LUMO further confirms that CA@PCN‐222 has a higher reduction ability, which facilitates its use as a promising photocatalyst (Figure 4i).

### 2.4 Photocatalytic CO_2_ Reduction Properties

**Figure 5:**
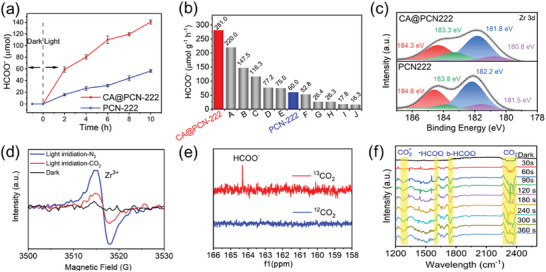
Photocatalytic performance and mechanism for CO_2_RR. a) Amounts of HCOO^−^ produced over time on CA@PCN‐222 and pristine PCN‐222. b) Photocatalytic efficiency of representative MOFs. (A: NNU‐29; B: MIL‐101 (Fe); C: NH_2_‐MIL‐101(Fe); D: Ru‐MOF; E: NH_2_‐MIL‐88B; F: NNU‐28; G: NH_2_‐UIO‐66; H: NNU‐31‐Zn; I: MOF‐253; and J: NH_2_‐MIL‐125‐Ti). c) In situ characterization of CA@PCN‐222. In situ high‐resolution XPS spectra of CA@PCN‐222 and PCN‐222 on irradiation at room temperature for Zr 3d. d) ESR spectra of CA@PCN‐222 under different conditions. e) ^13^C NMR spectra for the product obtained from reaction with red: ^13^CO_2_ or blue: ^12^CO_2_. f) In situ ATR‐FTIR spectra of CA@PCN‐222.

Given that the bandgap of CA@PCN‐222 was narrower than that of PCN‐222 and the LUMO band of CA@PCN‐222 exceeded the reduction potential of CO_2_ to C_1_ compounds and even reach the most negative reduction potential of formate (−0.28 V vs NHE), the photocatalytic CO_2_ reduction activities of CA@PCN‐222 were examined in CH_3_CN with TEOA at room temperature, which were used as an electron scavenger and solvent. The reduction in production activities is shown in **Figure** **5**a and Figure S25 (Supporting Information). Remarkably, CA@PCN‐222 exhibited significant photocatalytic activity for CO_2_RR. The resultant HCOO^−^ anion was detected through ion chromatography and the amount reached up to 2816.0 µmol g^−1^ in 10 h, which was >4 times higher than that of PCN‐222 (778.2 µmol g^−1^). Compared with most MOFs‐based photocatalysts (Figure 5b; Table S2, Supporting Information) used for photocatalytic CO_2_ reduction, for example, 1) 220.0 µmol g^−1^ h^−1^ for NNU‐29 (A), 2) 147.5 µmol g^−1^ h^−1^ for MIL‐101‐Fe (B), 3) 116.3 µmol g^−1^ h^−1^ for NH_2_‐MIL‐53‐Fe (C), 4) 77.2 µmol g^−1^ h^−1^ for Ru‐MOF (D), 5) 75.0 µmol g^−1^ h^−1^ for NH_2_‐MIL‐88B (E), 6) 52.8 µmol g^−1^ h^−1^ for NNU‐28 (F), and 7) 116.3 µmol g^−1^ h^−1^ for NH_2_‐MIL‐53‐Fe (G),CA@PCN‐222 (280.4 µmol g^−1^ h^−1^) exhibit higher performances.

Moreover, the amounts of by‐product CH_4_ and CO (Figures S26–S29 and Tables S3 and S4, Supporting Information) were extremely low with values of 0.007 and 0.062 µmol (0.012 and 0.045 µmol for PCN‐222) and those of H_2_ and O_2_ (Figures S28 and S29, Supporting Information) were not detected, indicating that the catalyst exhibits extremely high selectivity (≈99.9%). Recycling experiments (Figures S30 and S31, Supporting Information) demonstrated no noticeable change in the yield rate of HCOO^−^ occurred during the six reaction runs. Furthermore, the structure of CA@PCN‐222 remained almost intact after six reactions, as revealed from the SEM, XRD, XPS, EXAFS, and FT‐IR spectra data (Figures S32–S47, Supporting Information).

### 2.5 Mechanistic Investigations of Photocatalytic CO_2_ Reduction to Formate

**Figure 6:**
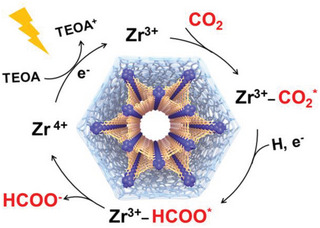
Proposed mechanism for the CO_2_ photoreduction reaction over CA@PCN‐222.

To elucidate the mechanism of the photocatalytic CO_2_ reduction to formate using CA@PCN‐222 as a catalyst, the reactants, and products were further determined. HCOO^−^ was not detected when the reaction was in the dark, and no HCOO^−^ was produced in the absence of either CA@PCN‐222 or triethanolamine (TEOA), indicating that these materials were necessary in the reaction. In situ irradiation XPS (Figure 5c; Figures S48–S50, Supporting Information) was used to explore the changing process of CA@PCN‐222 during the photocatalytic CO_2_ reduction reaction. In the dark, the Zr (IV) oxidation state in the Zr clusters was represented by the binding energies of Zr 3d_5/2_ and Zr 3d_3/2_ peaks of CA@PCN‐222, which were 183.1 and 185.5 eV, respectively. After irradiation, the Zr 3d_5/2_ band split into two peaks at 181.5 and 182.2 eV and Zr 3d_3/2_ band split into two peaks at 184.6 and 183.8 eV, suggesting a partial reduction of Zr (IV) to Zr (III). This further confirms that the photocatalytically active sites are located on the Zr clusters. Compared with PCN‐222, after irradiation, more negative shifts occurred in the four peaks of Zr 3d_5/2_ and Zr 3d_3/2_, indicating that more photoinduced electrons were transferred to the Zr clusters. Thus, Zr clusters with more negative binding energies have stronger CO_2_ reduction ability. This phenomenon is a result of the effect of the atomic interface on the MOFs. The binding energy shifts under dark and light conditions demonstrated more efficient charge transfer on CA@PCN‐222. This may be the result of an interfacial effect on the MOF. Electron paramagnetic resonance (EPR) was used to investigate the excited state of the CA@PCN‐222 sample (Figure 5d). Upon irradiation and surrounded by a N_2_ atmosphere, an obvious signal of Zr^III^ atoms was captured in CA@PCN‐222 samples corresponding to *g* = 2.002, which resulted from the photoexcited electron transfer. In addition, upon irradiation in a CO_2_ atmosphere, the signal of the Zr^III^ atoms in CA@PCN‐222 weakened significantly, indicating that the photogenerated electrons in the Zr^III^ ions migrated to CO_2_. Thus, these results demonstrate the electron transfer process in the photocatalytic CO_2_RR for CA@PCN‐222. Moreover, to identify the source of HCOO^−^, we utilized the isotope ^13^CO_2_ in the photocatalytic reaction process, which was identified using ^13^C NMR. The peak of HCOO^−^ at 164.8 ppm at the ^13^C NMR spectrum was collected, when ^13^CO_2_ was introduced (Figure 5e; Figure S51, Supporting Information). However, the signal of HCOO^−^ was not detected for ^12^CO_2_. In situ FTIR spectroscopy was employed to investigate the CO_2_ radicals and other reaction intermediates in the photocatalytic reaction (Figure 5f; Figure S52, Supporting Information). No discernible changes in the vibrational peaks were observed in the in situ FTIR spectra of CA@PCN‐222 in a dark CO_2_ atmosphere. The peaks from 2500 to 2400 cm^−1^ correspond to CO_2_ vibration. By contrast, the peaks at 1273, 1600, and 1740 cm^−1^ appeared after irradiation. As illustrated in Figure 5f, the signals arose at 1273 and 1600 cm^−1^ and grew stronger over time as the irradiation time increased. The characteristic peak at 1740 cm^−1^ was attributed to the formation of bidentate formate, whereas the peak at 1395 cm^−1^ indicated the formation of ^*^HCOO. Hence, the proposed mechanism for CO_2_ photoreduction reaction over CA@PCN‐222 is shown in **Figure** **6**.

The doping of cellulose acetate has a significant regulatory effect on zirconium‐based MOF. In order to determine whether similar modulatory effects are observed for other polyvalent metal ions MOFs, we again tried several other metals MOFs using the method elaborated in this paper, including Cu‐based MOF (HKUST‐1), Co‐based MOF (Zif‐67), Ti‐based MOF (MIL‐125), Fe‐based MOF (MIL‐53), and other two Zr‐based MOFs (PCN‐138 and NH_2_‐UiO‐66). The XRD of those MOF as well as the MOF complexes with cellulose acetate were tested as shown in Figure S53 (Supporting Information). The results indicate that these MOFs and CA@MOFs are successfully prepared. We also tested their XANES in Figure S54 (Supporting Information), indicating that those metal valence states of CA@MOFs are lower, and the strategy is also applicable to other polyvalent metal ions MOF. The TEM photos of those were displayed in Figures S55–S60 (Supporting Information), and their morphologies have changed differently after the addition of cellulose acetate. In addition, we also did further photocatalytic experiments for CA@MOFs and MOFs in Figure S61 (Supporting Information). The results showed that the photocatalytic activity of CA@MOFs increased to varying degrees, except for CA@ZiF‐8 and CA@HKUST‐1.

### 2.6 Theoretical Calculations

**Figure 7:**
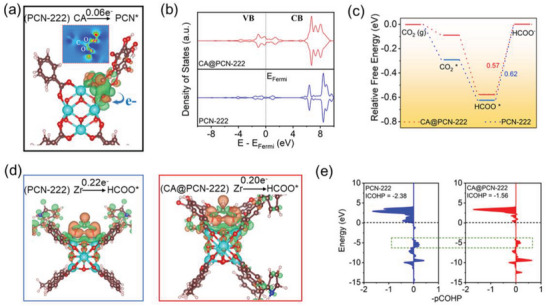
Theoretical CO_2_RR activity of CA@PCN‐222. a) Diagram of the charge density difference between PCN and CA, b) pDOS of Zr‐d orbitals for PCN‐222 and CA@PCN‐222. c) Free energy profile of CO_2_RR to HCOO^−^ on different models. d) Diagram of the charge density difference between different models and adsorbed HCOO^*^ intermediates. e) Calculated projected crystal orbital Hamilton population (pCOHP) diagram of Zr‐HCOO^*^ interaction.

As shown in **Figure** **7**a, the charge density differences of CA@PCN‐222 exhibited interfacial electron transfer (ca. 0.06 e^−^) from CA to Zr‐oxo, which is consistent with our XPS and XAS results. This led to redistribution of Zr d‐orbital electrons. The projected density of states (pDOS) diagrams in Figure 7b revealed that the valence band (VB) of CA@PCN‐222 widens and crosses the Fermi level (*E*_Fermi_) compared with that of the parent PCN‐222, which contributes to higher charge‐transfer kinetics.^[^ ^16^ ^]^

To explore the origin of the catalytic activity of the PCN‐222 and CA@PCN‐222 catalysts, the free energy diagram of the CO_2_RR to HCOO^−^ was calculated, as illustrated in Figure 7c, and the related intermediate structures are shown in Figures S62 and S63 (Supporting Information). The results showed that the CO_2_^*^ and HCOO^*^ formation processes for the investigated models were energy downhill, indicating that CO_2_ adsorption and protonation were spontaneous. This was because the positively charged Zr‐oxo sites (i.e., Lewis acids) could effectively benefit the process of CO_2_ (as a typical Lewis base) adsorption and activation. Notably, the desorption process of HCOO^*^ acts as the rate‐determining step for investigated models (Figures S64–75, Supporting Information), in which the energy barrier for CA@PCN‐222 composite is 0.53 eV, which is much <0.62 eV for PCN‐222. This improves the catalytic activity of CA@PCN‐222 by accelerating the rate‐limiting step.

Because HCOO^*^ desorption acts as the rate‐determining step in the present study, the charge density differences for HCOO^*−^ adsorbed coordination were plotted to analyze the effect of CA on charge transfer between the catalyst and the HCOO^*^ intermediate. The results in Figure 7d show that for the CA@PCN‐222 composite, fewer electrons can be transferred from the Zr‐oxo reactive sites to the HCOO^*^ species, which corresponds to a weaker covalent Zr‐HCOO^*^ interaction after CA treatment. The projected crystal orbital Hamilton population (pCOHP) was calculated to clarify the interaction between the Zr‐oxo sites and adsorbed HCOO^*^.^[^ ^18^ ^]^ The positive values in the pCOHP diagram represent bonding contributions and the negative values represent antibonding contributions. The results in Figure 7e show that the antibonding contributions of Zr‐HCOO^*^ interactions are largely weakened for CA@PCN‐222. As a result, the CA@PCN‐222 composite exhibited a smaller ICOHP value (−1.56, calculated as the energy of the pCOHP integral up to the Fermi level) than PCN‐222, which further revealed that CA treatment contributes to HCOO^*^ desorption, thus exhibiting superior CO_2_RR to HCOO^−^ activity.

**Figure 8:**
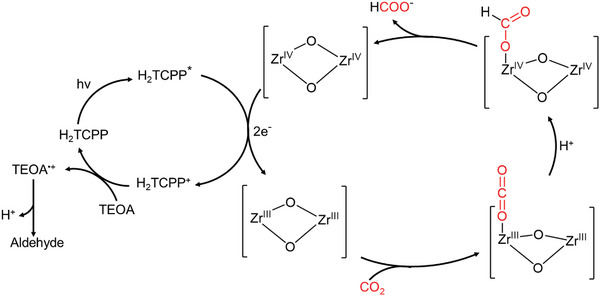
Proposed catalytic mechanism of photocatalytic CO_2_ reduction to HCOO^−^. The photo‐initiated electron transfers from H_2_TCPP to Zr‐clusters in CA@PCN‐222 lead to the photo‐reduction of CO_2_.

The detailed mechanism of CO_2_ photoreduction based on CA@PCN‐222 should be described in **Figure** **8**. In light of the above experimental results and DFT calculations, the photocatalytic mechanism is proposed. Under the visible‐light irradiation, the H_2_TCPP ligand will be excited to excited state H_2_TCPP^*^, which can transfer one electron to the Zr^IV^‐cluster unit through multistep relaxation. Consequently, the Zr^IV^‐cluster unit accepted two electrons from two adjacent H_2_TCPP ligand to give Zr^III^‐cluster active site, which can selectively reduce CO_2_ to HCOO^−^ in a two‐electron process. Finally, the H_2_TCPP^+^ can be reduced back to the H_2_TCPP by sacrificial donor (TEOA) to complete the catalytic cycle.

## 3 Conclusion

In this study, we rationally designed cellulose acetate@PCN‐222 hybrids with strong electronic coupling using a practical strategy in which CA was grown in situ on the surface of PCN‐222. The interface effects were appropriately monitored using comprehensive synchrotron radiation‐based X‐ray spectroscopy and transmission electron microscopy. The highly correlated nanocable‐like heterogeneous structures established effective charge‐transfer channels and active sites on PCN‐222. The novel CA@PCN‐222 photocatalyst exhibited remarkable performance in the photocatalytic CO_2_ reduction to formate. We expect that the interface electronic strategy will provide a basic idea for guiding the researchers regarding the investigation of advanced photocatalysts with high efficiency.

## 4 Experimental Section

### Synthesis of PCN‐222

A mixture of ZrCl_4_ (75 mg, 0.322 mmol), H_4_L (192 mg, 0.243 mmol), and formic acid (19.2 mL) was dissolved in *N*,*N*‐dimethylFormamide (DMF) (36 mL) and stirred for 30 min at room temperature. The mixture was then transferred to 12 Teflon autoclaves (20 mL) and heated at 100 °C for 2 days to obtain purple samples. After washing with DMF and acetonitrile for several times, the synthesized samples were dried at 200 °C for eliminating formates.^[^ ^19^ ^]^

### Synthesis of CA@PCN‐222 Composite

A mixture of ZrCl_4_ (75 mg, 0.322 mmol), H_4_L (192 mg, 0.243 mmol), CA (240 mg, 0.0024 mmol), and formic acid (19.2 mL) was dissolved in DMF (36 mL) and stirred for 30 min at room temperature. The mixture was then transferred to 12 Teflon autoclaves (20 mL) and heated at 100 °C for 2 days to obtain purple samples. After washing with DMF and acetonitrile for several times, the synthesized samples were dried at 200 °C for eliminating formats.

### Electrochemical Characterization

The working electrodes were prepared by applying 15 µL of slurry to the surface of fluoride‐tin oxide (FTO) glass plates and covering ≈1 cm^2^. The slurry was prepared by mixing 0.02 g of sample and 2 mL of ethanol. Electrochemical measurements were conducted using a BAS Epsilon Electrochemical System in a conventional three‐electrode cell using a Pt plate as the counter electrode and an Ag/AgCl electrode (3 m KCl) as the reference electrode. The experiments were performed at room temperature in a 0.2 m Na_2_SO_4_ electrolyte deoxygenated using an N_2_ stream.

### Photocatalytic Reaction

Photocatalyst (50 mg) was added to a MeCN and TEOA solution (60 mL, V/V:30/1) where the mixed solution was pre‐degassed with CO_2_ to remove the dissolved O_2_ for 30 min. And then, the solution was injected with CO_2_ for 15 min and maintained under a carbon dioxide atmosphere at ambient pressure. Subsequently, a 300 W Xe lamp was used in the photocatalytic CO_2_ reduction reaction, where the UV‐cut filter and IR‐cut filter were involved to remove light with wavelengths <400 nm and longer than 800 nm. The optical power density illuminated on the reaction container was 180 mW cm^−2^. The solution of the reaction system was withdrawn and filtered using a filtration membrane (0.22 µm) to separate photocatalyst at a given time. The products in the liquid phase were analyzed using the ICS 940 (Metrohm), where the eluent consists of 1.0 mm NaHCO_3_ and 3.2 mm Na_2_CO_3_. Although alcohol could not be detected using the IC method, the reaction solution was examined using the flame ionization detector (FID) of a gas chromatograph (GC, Agilent), and no signal for alcohols were observed. In addition, this study also attempted to detect H_4_, CO, and H_2_ using both the FID (for detecting CH_4_ and CO; CO will be converted to CH_4_ using a methanation reactor prior to detection) and a thermal conductivity detector (TCD, for detecting H_2_) of a GC (GC2014, Ar carrier, Shimadzu). No signals corresponding to H_2_, CH_4_, or CO were observed.

### In Situ IR Spectroscopy

Before the IR measurements, the samples were soaked in anhydrous MeCN, and the solvents were exchanged every 12 h. The samples were then dried under vacuum at 100 °C for several hours in Schlenk tubes. The Nicolet iS50 instrument equipped with an MCT detector was used to collect the transmission IR spectra of powder samples at a 4 cm^−1^ resolution. Approximately 50 mg of the solid sample was placed in an in situ IR cell in a glovebox. The cell was filled with CO_2_ and TEOA and the spectra were recorded without exposing the sample to air.

### X‐Ray Absorption Fine Structure Measurements

The Zr K‐edge X‐ray absorption data were collected from the BL1W1B station in the Beijing Synchrotron Radiation Facility (operated at 2.5 GeV with a maximum current of 250 mA). The radiation was monochromatized using a Si (111) double‐crystal monochromator. The intensity of the incident X‐rays was monitored using an Ar‐filled ion chamber (*I*_0_) placed in front of the sample. The solid samples were placed in an aluminum sample holder sealed with Kapton tape. Fluorescence excitation spectra data were collected using a Lytle detector. The energy was calibrated using the first peak maximum of the first derivative for the Zr foil. The reference spectra were recorded in the transmission mode using an Ar‐filled ionization chamber. All the data were collected at room temperature.

## Conflict of Interest

The authors declare no conflict of interest.

## Supporting information

Supporting Information

## Data Availability

The data that support the findings of this study are available in the supplementary material of this article.
