# Supplementary material for: Enhancing the Photocatalytic Activity of Zirconium‐Based Metal–Organic Frameworks Through the Formation of Mixed‐Valence Centers
Source: Adv Sci (Weinh). 2023 Aug 7;10(29):2303206. doi: 10.1002/advs.202303206 (PMC10582444; doi:10.1002/advs.202303206)
Supplement: Supplementary file 1 — Supporting Information [file ADVS-10-2303206-s001.pdf]

ADVANCED SCIENCE
Open Access

Supporting Information

for *Adv. Sci.*, DOI 10.1002/advs.202303206

Enhancing the Photocatalytic Activity of Zirconium-Based Metal–Organic Frameworks Through the Formation of Mixed-Valence Centers

Zihao Wei, Shaojia Song, Hongfei Gu, Yaqiong Li, Qi Sun, Ning Ding, Hao Tang, Lirong Zheng, Shuhu Liu, Zhenxing Li, Wenxing Chen, Shenghua Li* and Siping Pang**

Supporting Information

Enhancing the Photocatalytic Activity of Zirconium-Based Metal–Organic Frameworks Through the Formation of Mixed-Valence Centers

*Zihao Wei, Shaojia Song, Hongfei Gu, Yaqiong Li, Qi Sun, Ning Ding, Hao Tang, Lirong Zheng, Shuhu Liu, Zhenxing Li, * Wenxing Chen, Shenghua Li, * Siping Pang**

Experimental Section

Materials and Equipment.

H_2TCPP (tetrakis (4-carboxyphenyl)-porphyrin) was from Shang Hai Tensus Biotech Co., Ltd. Zirconium (IV) chloride ($ZrCl_4$) and cellulose acetate were from TCI. N, N-dimethylformamide (DMF), acetonitrile (CH_3CN), triethanolamine (TEOA), benzoic acid, formic acid, and sodium sulfate (Na_2SO_4) were purchased from Sinopharm Chemical Reagent Co., Ltd. All commercial chemicals were used without further purification unless otherwise mentioned.

All the reagents were analytical, and all the glassware apparatuses were washed with alkali liquor and deionized water. Powder X-ray diffraction (XRD) patterns were obtained on a diffractometer (Cu Kα radiation, λ=0.15417 nm) with 2θ ranging from 5 to 40°. Fourier transform infrared (FT-IR) spectra were recorded on a Spectrum One in the spectral range of 400 cm^{-1} – 4000 cm^{-1} using the KBr disk method with a PerkinElmer instrument. Thermogravimetric analysis (TGA) data were collected using a Labsys evo system at a heating rate of 10 K min^{-1} from room temperature to 800 ^{O} C under atmosphere. Fluorescence spectra were recorded on the LS – 55 spectrophotometer at room temperature. Fluorescence lifetime measurements were conducted using FLS980 spectrophotometer at room temperature. UV-vis. spectra were recorded on Shimadzu UV 3600 plus spectrophotometer. The valence state of chemical element bound to photocatalysis was analyzed based on X-ray Photoelectron Spectroscopy (XPS) spectra (Thermo Fisher Scientific, UK) Photocurrent measurements and electrochemical impedance spectroscopy were conducted on electrochemical workstation CHI 660E (ChenHua Instrument, Shanghai). In-situ XPS was measured by Thermo SCIENTIFIC SCALAB 250Xi.

$^{13}CO_2$ isotope tracking experiment

First, 25 ml reaction flask was sealed by a counter-ported rubber plug, and was pumped vacuum. The mixed solution of 3ml CD_3CN and 2.5mg catalyst was injected into the reaction flask, and was frozen with liquid N_2. Meanwhile, the reaction flask was pumped for 5minutes. And then, wait for them to melt, along with bubbles popping up. Repeat the above operation several times until no more air bubbles were discharged. Then, 0.1 ml TEOA was was injected into the reaction flask, and repeat the above exhaust process several times until no more air bubbles were discharged. Finally, pass $^{13}CO_2$ into the reaction flask. The solution was irradiated with a 300 W Xe lamp for 10 hours with a UV-cut filter to remove light with wavelengths less than 400 nm and an IR-cut filter to remove wavelengths longer than 800 nm. After the reaction, the filtrate was filtered using a filter membrane for ^{13}C NMR.

XAFS data processing

The acquired EXAFS data were processed according to the standard procedures using the Athena and Artemis implemented in the IFEFFIT software packages. The fitting detail is described below:

The acquired EXAFS data were processed according to the standard procedures using the ATHENA module implemented in the IFEFFIT software packages. The EXAFS spectra were obtained by subtracting the post-edge background from the overall absorption and then normalizing with respect to the edge-jump step. Subsequently, the $\chi(k)$ data of were Fourier transformed to real (R) space using a hanning windows (dk=1.0 Å^{-1}) to separate the EXAFS contributions from different coordination shells. To obtain the quantitative structural parameters around central atoms, least-squares curve parameter fitting was performed using the ARTEMIS module of IFEFFIT software packages.

The following EXAFS equation was used:

$$\chi(k) = \sum_j \frac{N_j S_o^2 F_j(k)}{k R_j^2} \exp[-2k^2\sigma_j^2] \exp[\frac{-2R_j}{\lambda(k)}] \sin[2kR_j + \phi_j(k)]$$

S_0^2 is the amplitude reduction factor, $F_j(k)$ is the effective curved-wave backscattering amplitude, N_j is the number of neighbors in the jth atomic shell, R_j is the distance between the X-ray absorbing central atom and the atoms in the jth atomic shell (backscatterer), λ is the mean free path in Å, $\phi_j(k)$ is the phase shift (including the phase shift for each shell and the

total central atom phase shift), σ_j is the Debye-Waller parameter of the jth atomic shell (variation of distances around the average R_j). The functions $F_j(k)$, λ and $\phi_j(k)$ were calculated with the ab initio code FEFF8.2. The coordination numbers of model samples were fixed as the nominal values. The obtained S_0^2 was fixed in the subsequent fitting. While the internal atomic distances R, Debye-Waller factor σ^2, and the edge-energy shift ΔE_0 were allowed to run freely.

Computational Method

All density functional theory (DFT) calculations were carried out within the Vienna ab initio simulation program package (VASP 5.4.4) using the GGA-PBE electron exchange-correlation functional. The cutoff energy was set to 400 eV, and Brillouin zone sampling was Gamma point. The Zr–oxo ($C_{70}H_{54}O_{24}Zr_6N_3$) with a linker defect is established to describe the parent PCN222 model [1], in which CO_2 and the reaction intermediate species are adsorbed on two adjacent open Zr sites. Then, the cellulose acetate (CA) modified Zr-oxo model was used to model the CA @ PCN222 composite. Geometry optimization was carried out with the force criterion < 0.02 eV/Å. The atoms in the unit cell were allowed to relax, while the lattice constants were kept fixed [2]. Gibbs free energies for gaseous and adsorbed species were calculated at 298.15 K via VASPKIT program, according to the expression [3]:

$$G = EDFT + EZPE – TS$$

The free energy of the ($H^+ + e^-$ pair) is related to that of 1/2 H_2 in the gas-phase [4].

The calculated elementary steps include [5]:

(1) slab* + CO_2 (g) → CO_2*

(2) CO_2* + H^+ + e^- → HCOO*

(3) HCOO * → $HCOO^-$ + slab*

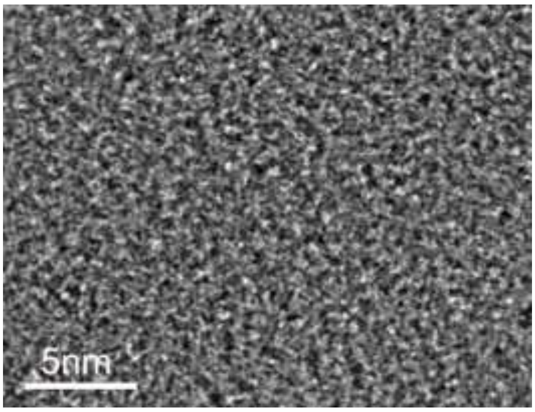

Figure S1 HAADF images of CA@PCN-222.

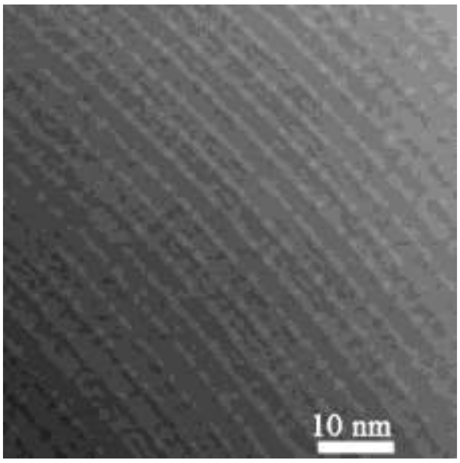

Figure S2 HAADF images of pristine PCN-222.

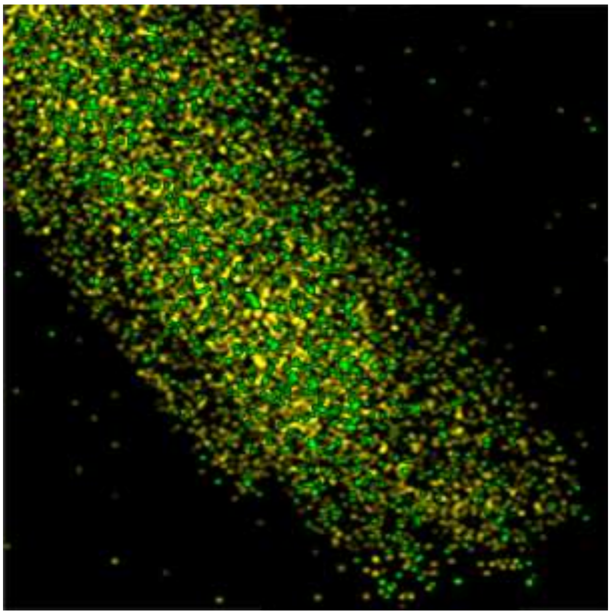

Figure S3 Superimposed energy spectra of Zr and C of CA@PCN-222.

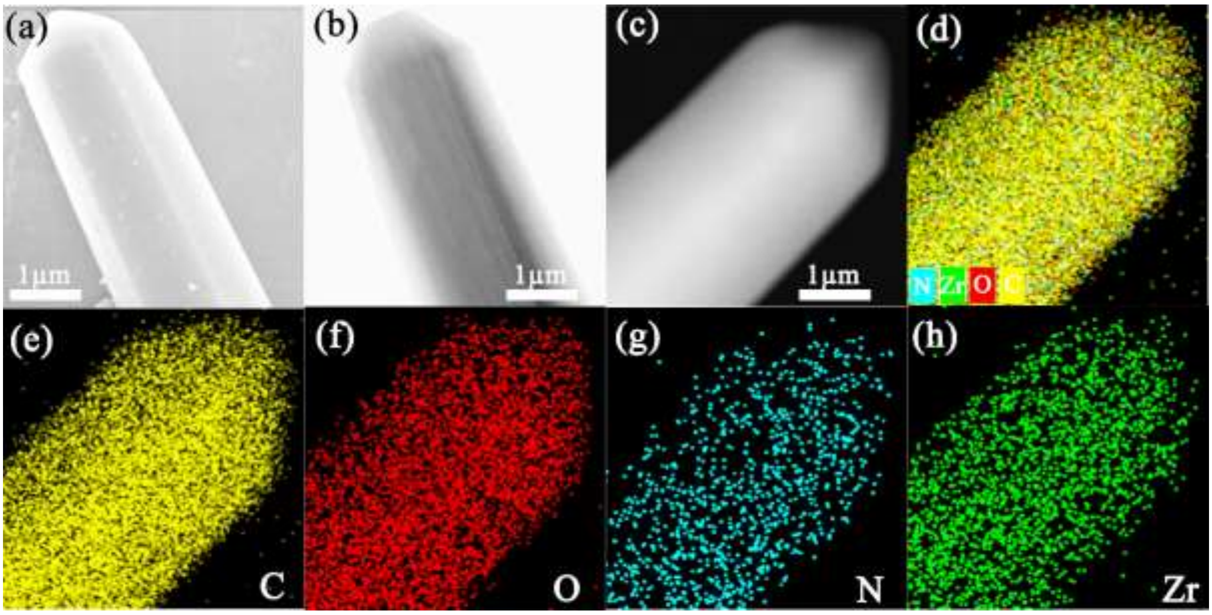

Figure S4 TEM image, HAADF image and EDX mapping of pristine PCN-222.

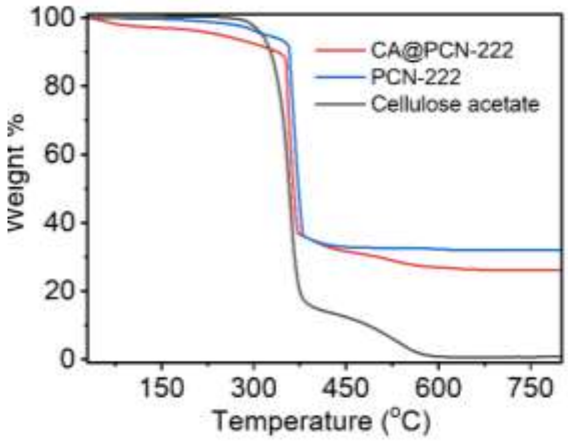

Figure S5 TG curves of CA@PCN222, pristine PCN222 and Cellulose acetate.

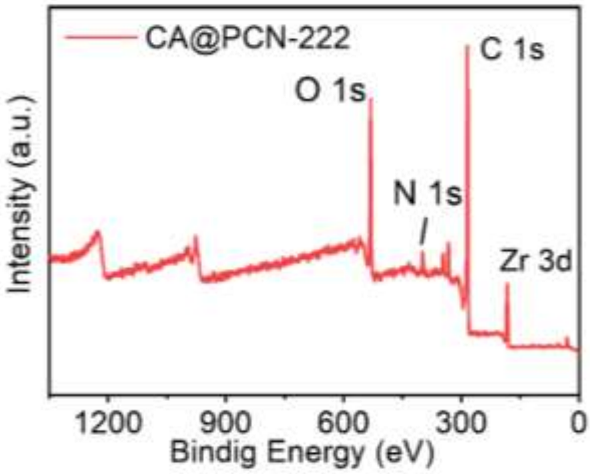

Figure S6 XPS survey spectrum of CA@PCN222.

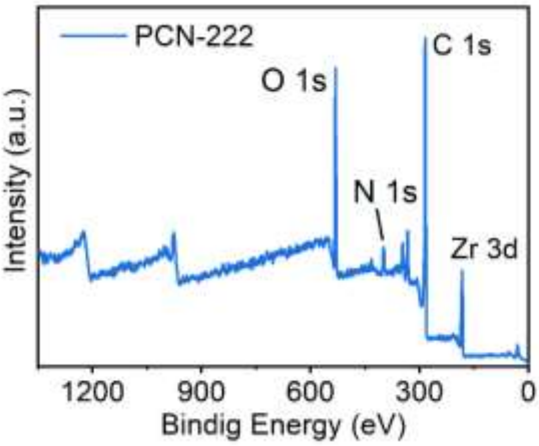

Figure S7. XPS survey spectrum of pristine PCN222.

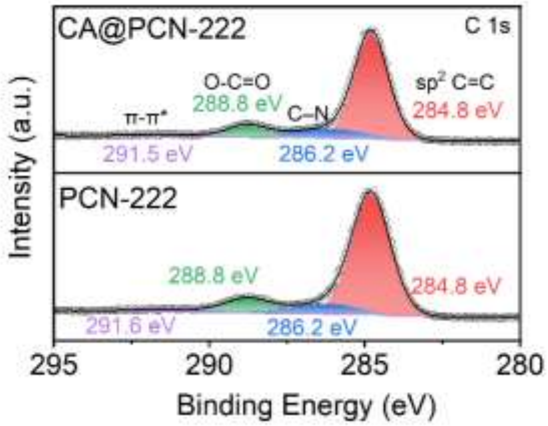

Figure S8. High-resolution XPS spectra of C 1s region for CA@PCN-222 and pristine PCN-222.

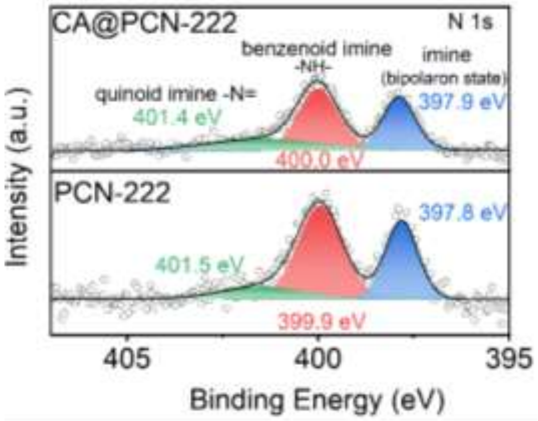

Figure S9. High-resolution XPS spectra of C 1s region for CA@PCN-222 and pristine PCN-222.

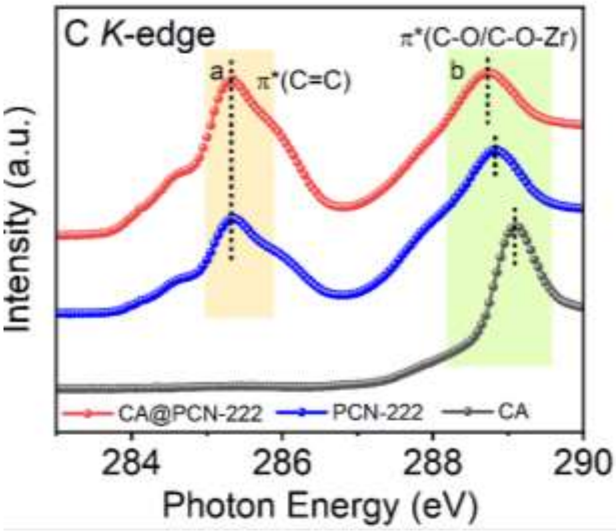

Figure S10. C K-edge XANES spectra of the CA@PCN-222, pristine PCN-222 and ZrO_2.

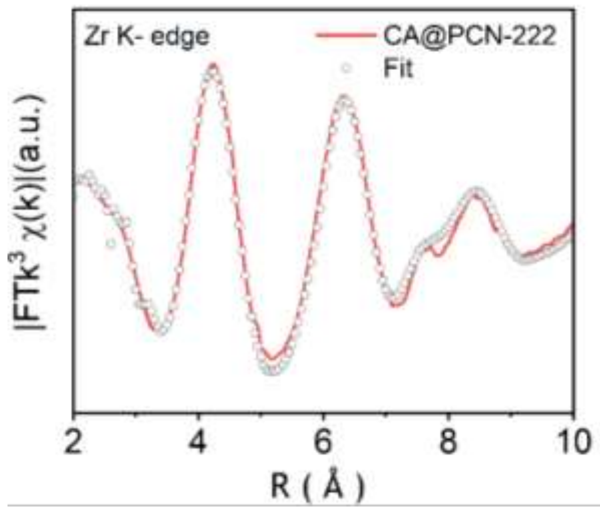

Figure S11. k3-weighted Zr K-edge EXAFS spectra of pristine CA@PCN-222.

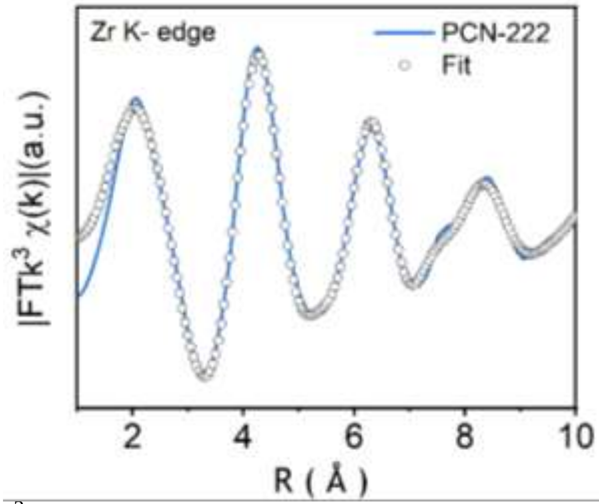

Figure S12. k^3-weighted Zr K-edge EXAFS spectra of pristine PCN-222.

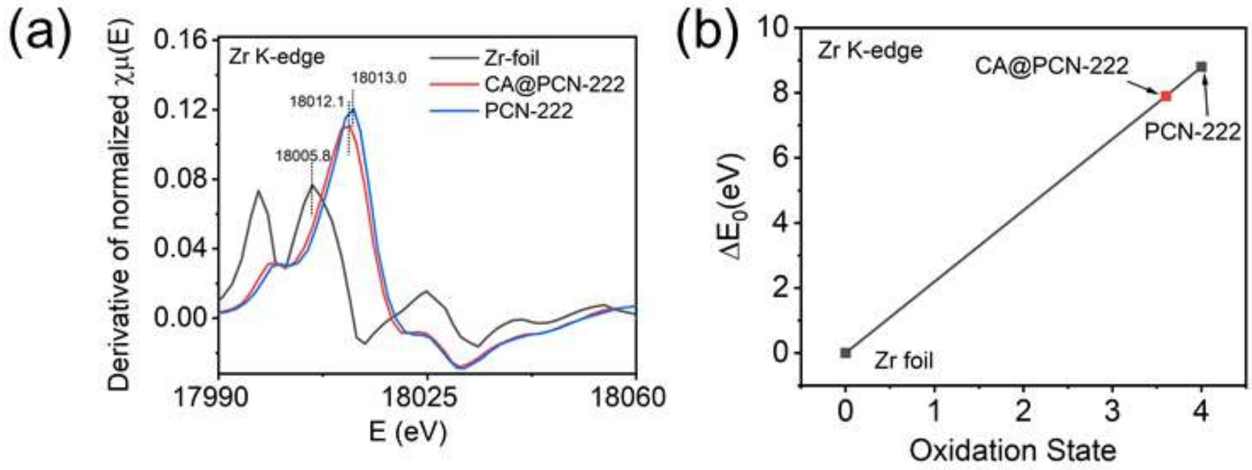

Figure S13. (a) First-derivative XANES curves of CA@PCN-222 and pristine PCN-222 for Zr K-edge. (b) Oxidation state of CA@PCN-222 and pristine PCN-222 for Zr K-edge.

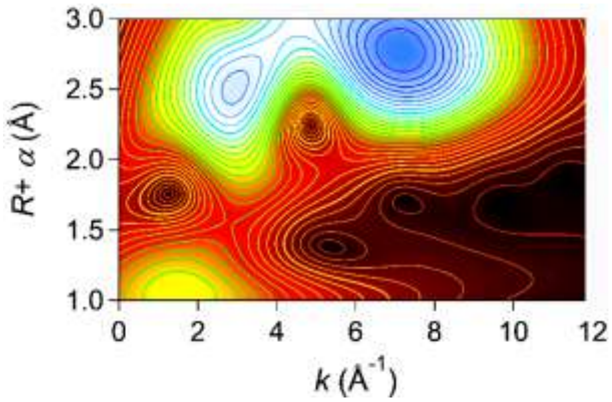

Figure S14. WT-EXAFS plots of Zr foil.

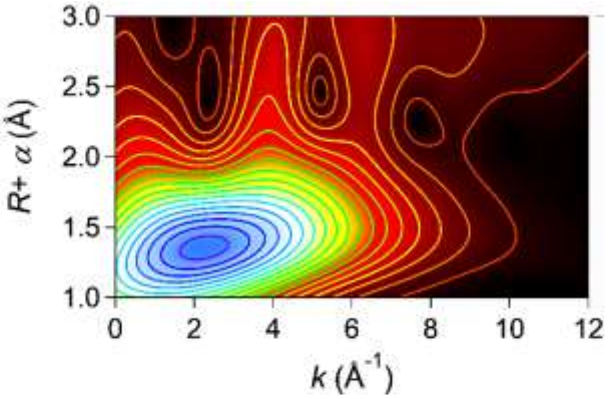

Figure S15. WT-EXAFS plots of ZrO_2.

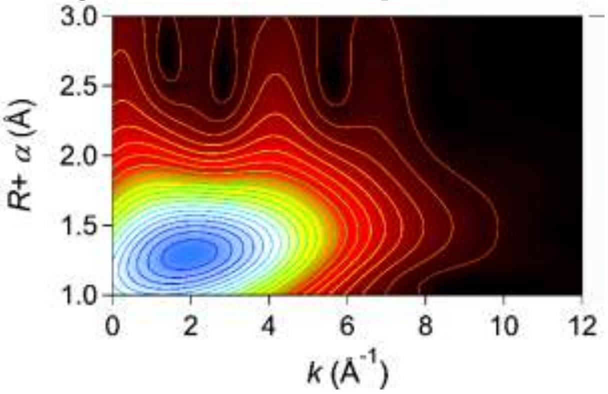

Figure S16. WT-EXAFS plots of CA@PCN-222.

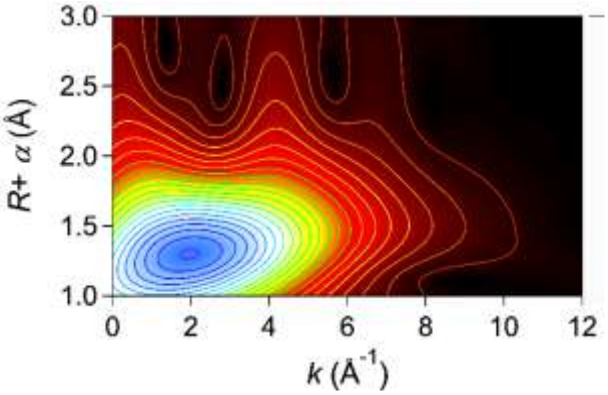

Figure S17. WT-EXAFS plots of PCN-222.

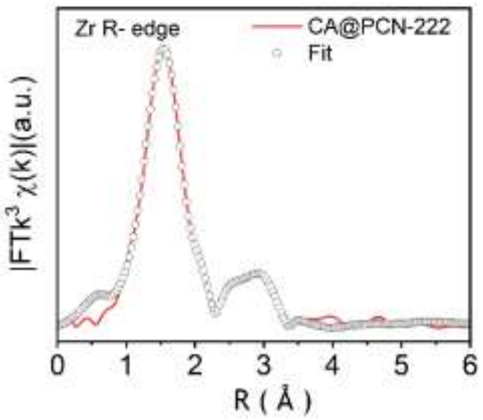

Figure S18. EXAFS fitting curves of CA@PCN-222 at Zr K-edge in R space.

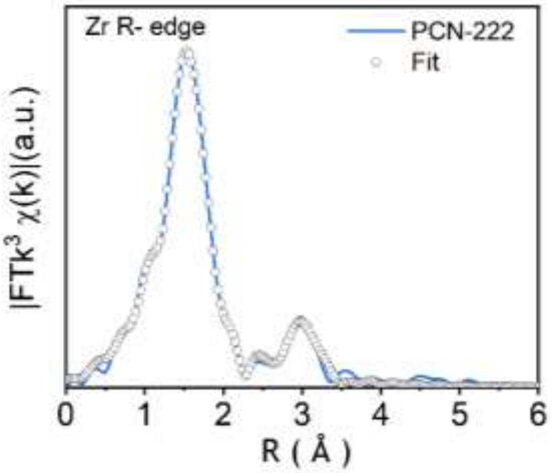

Figure S19. FT k^3-weighted Zr K-edge EXAFS spectra of pristine PCN-222.

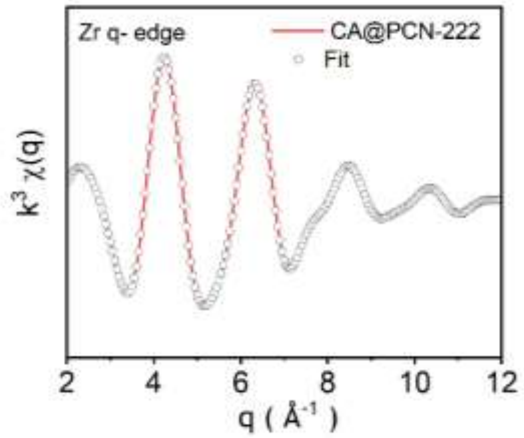

Figure S20. k^3-weighted Zr q space EXAFS fitted curve of CA@PCN-222.

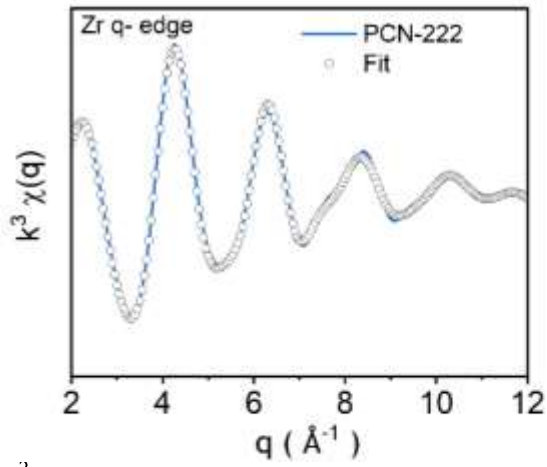

Figure S21. k^3-weighted Zr q space EXAFS fitted curve of PCN-222.

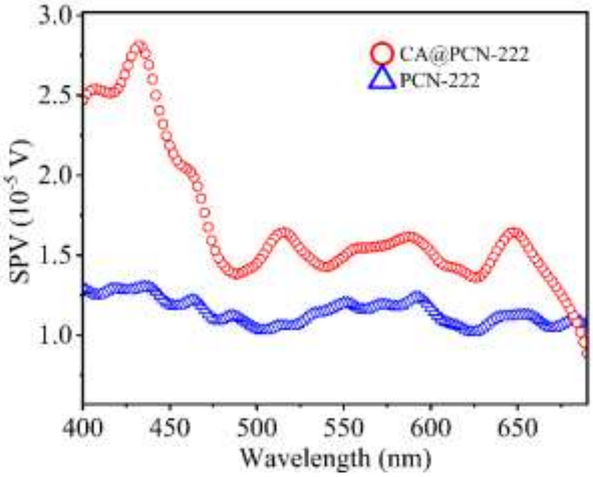

Figure S22. Steady-state surface photovoltage spectrum of CA@PCN-222 and pristine PCN-222.

Table S1. Structural parameters extracted from the Zr K-edge EXAFS fitting. (S_0^2=0.85).

Sample	Scattering pair	CN	R(Å)	$\sigma^2(10^{-3}Å^2)$	ΔE_0(eV)	R factor
CA@PCN-222	Zr-O	5.8	1.92	4.5	1.5	0.007
	Zr-Zr	3.2	3.70	5.9	1.5	
PCN-222	Zr-O	6.0	1.95	4.7	2.0	0.008
	Zr-Zr	3.5	3.72	4.6	2.0	

S_0^2 is the amplitude reduction factor; CN is the coordination number; R is interatomic distance (the bond length between central atoms and surrounding coordination atoms); σ^2 is Debye-Waller factor (a measure of thermal and static disorder in absorber-scatterer distances); ΔE_0 is edge-energy shift (the difference between the zero kinetic energy value of the sample and that of the theoretical model). R factor is used to value the goodness of the fitting. Error bounds that characterize the structural parameters obtained by EXAFS spectroscopy were estimated as N ± 20%; R ± 1%; σ^2 ± 20%; ΔE_0 ± 20%.

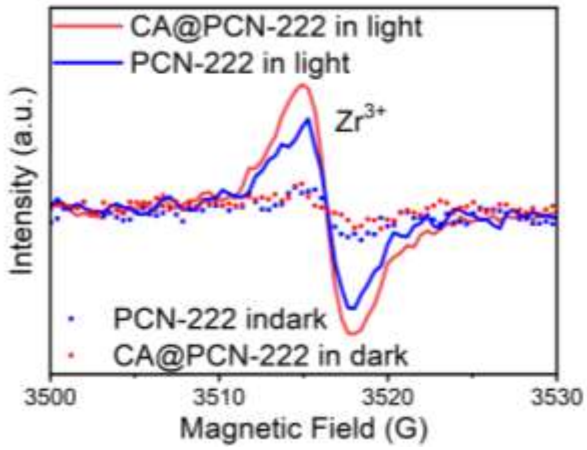

Figure S23. ESR spectra of CA@PCN-222 and PCN-222 under different conditions.

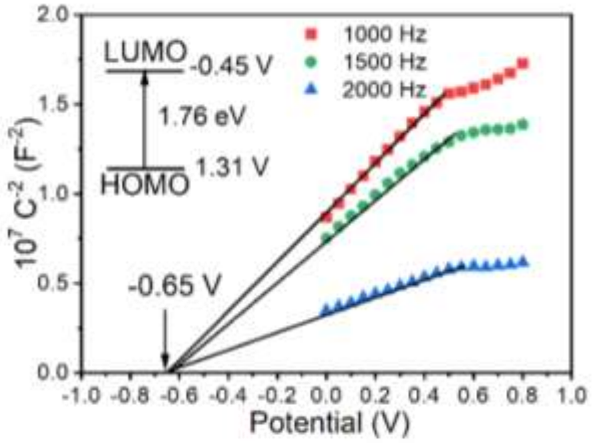

Figure S24. Mott-Schottky curve of PCN-222 at 1000 Hz, 1500 Hz, 2000 Hz.

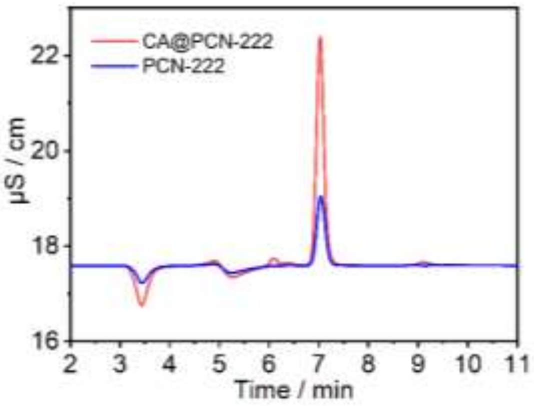

Figure S25. The ion chromatography traces of $HCOO^-$. The red and blue curve is for CO_2 photoreduction by CA@PCN-222 and pristine PCN-222 at 10h, respectively.

Table S2. Comparison of the photocatalytic performance of **CA @ PCN-222**, PCN-222, and reported crystalline photocatalysts for CO_2-to-$HCOO^-$ conversion under visible light irradiation.

Photocatalysisi	Solvent	Light source	$HCOO^-$ (μmol/g/h)	Reference
CA @ PCN-222	MeCN	300 w Xe-lamp, 400 – 800 nm	280.4	This work
PCN-222	MeCN	300 w Xe-lamp, 400 – 800 nm	76.3	This work
PCN-222	MeCN	300 w Xe-lamp, 420 – 800 nm	60	(6)
NH_2-MIL125-Ti	MeCN	300 w Xe-lamp, 420 – 800 nm	16.3	(7)
MOF-253	MeCN	300 w Xe-lamp, 420 – 800 nm	17.8	(8)
NH_2-UIO-66	MeCN	300 w Xe-lamp, 420 – 800 nm	26.4	(9)
NNU-28	MeCN	300 w Xe-lamp, 420 – 800 nm	52.8	(10)
NH_2-MIL-88B	MeCN	300 w Xe-lamp, 420 – 800 nm	75.0	(11)
NH_2-MIL-53 (Fe)	MeCN	300 w Xe-lamp, 420 – 800 nm	116.3	(12)
MIL-101 (Fe)	MeCN	300 w Xe-lamp, Visible light	147.5	(13)
NH_2-MIL-101 (Fe)	MeCN	300 w Xe-lamp, 420 – 800 nm	445.0	(13)
NNU-29	H_2O	300 w Xe-lamp, 420 – 800 nm	220.0	(14)
NNU-31-Zn	H_2O	300 w Xe-lamp, 420 – 800 nm	26.3	(15)
Ru-MOF	MeCN		77.2	(16)

Calculation method of GC product yield.

The yield of CH_4 and CO is calculated using the following method. First, we made the standard curves of CH_4 and CO by GC. As shown in the Figure S26 and S27, the X-axis is the mass (m) of CH_4/CO and the Y-axis is the peak area (μV*s). The CH_4 standard curve was obtained by fitting: $A = 755.455627 + 2551.264746 \times m$. And the CO standard curve was obtained by fitting: $A = -5450.218750 + 9219.592773 \times m$. The Raw data of the gaseous reaction products for CA@PCN-222 and PCN-222 of GC-FID showed in Figure S28 and S29. Calculated by the two equations above, the amounts of by-product CH_4 and CO for CA@PCN-222 were extremely low with values of 0.007 and 0.062 μmol. And the amounts of by-product CH_4 and CO for PCN-222 were 0.012 and 0.045 μmol.

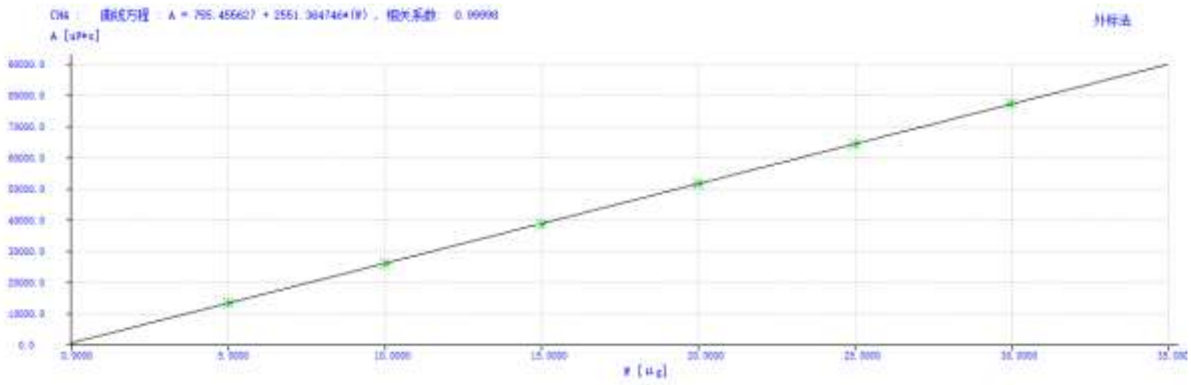

Figure S26 The CH_4 standard curve for GC-FID.

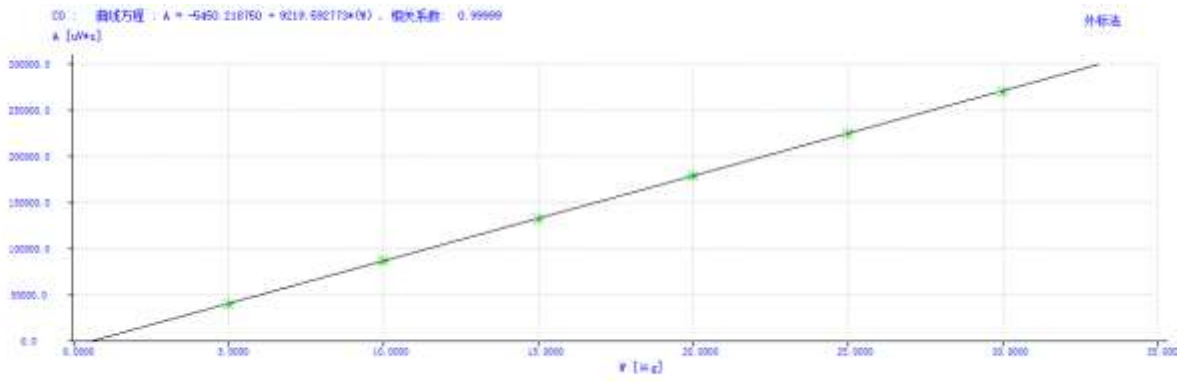

Figure S27 The CO standard curve for GC-FID.

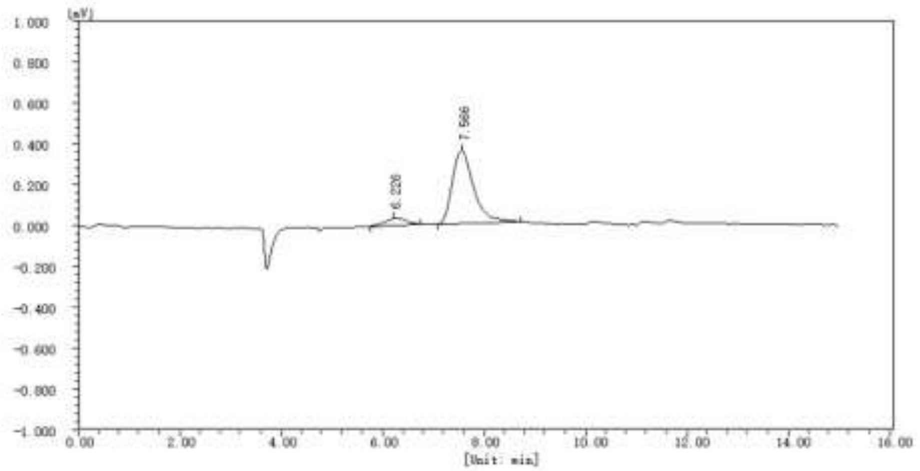

Figure S28 The raw data of the gaseous reaction products for CA@PCN-222 of GC-FID.

Table S3 Analysis results of GC for CA@PCN-222.

components	Retention time (min)	Half-peak width (min)	Peak High (μV)	Peak area (μV*s)
CH_4	6.226	0.487	36.9	1047.7
CO	7.566	0.435	359.9	10563.1

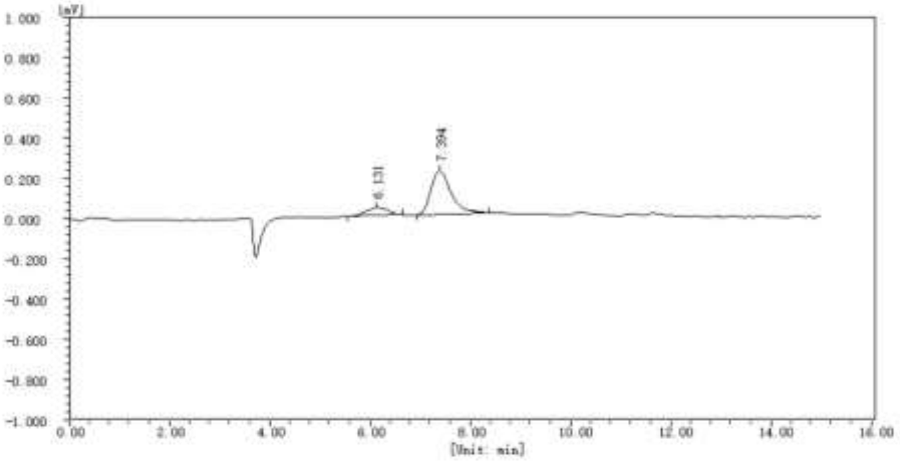

Figure S29 The raw data of the gaseous reaction products for PCN-222 of GC-FID.

Table S4. Analysis results of GC for PCN-222.

components	Retention time (min)	Half-peak width (min)	Peak High (μV)	Peak area (μV*s)
CH_4	6.131	0.495	40.8	1242.9
CO	7.394	0.414	220.8	6190.3

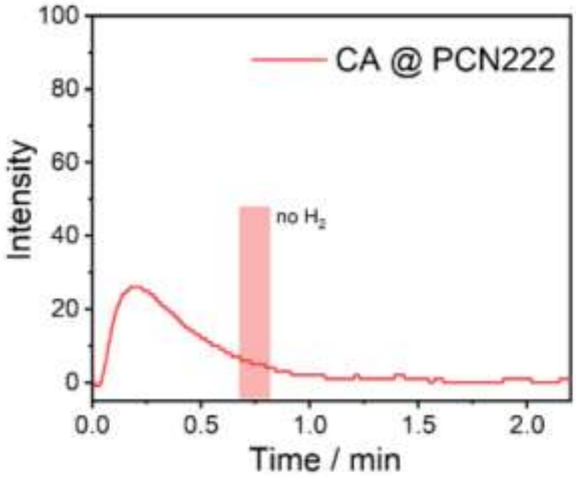

Figure S30. GC analysis of the gaseous reaction products for CA@PCN-222 by using the flame ionization detector (TCD).

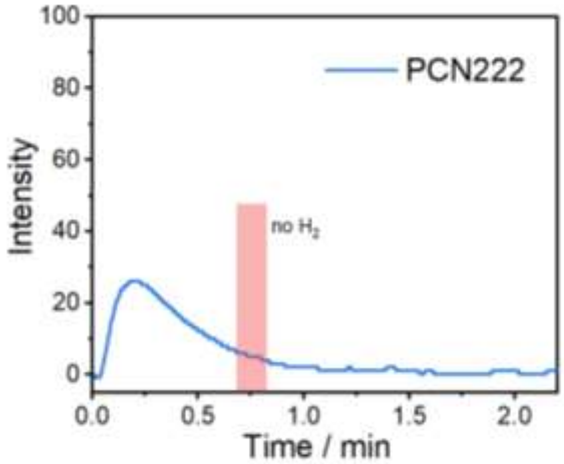

Figure S31. GC analysis of the gaseous reaction products for pristine PCN-222 by using the flame ionization detector (TCD).

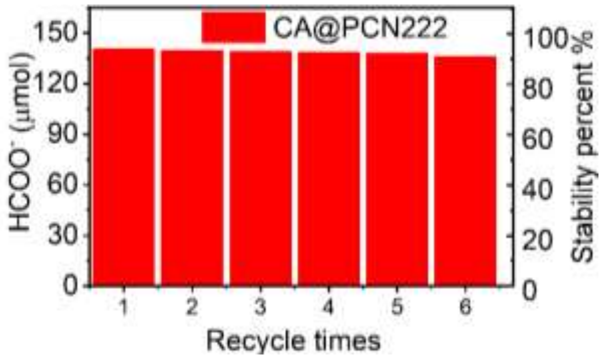

Figure S32. Generation of $HCOO^-$ in stability tests for CA@PCN-222.

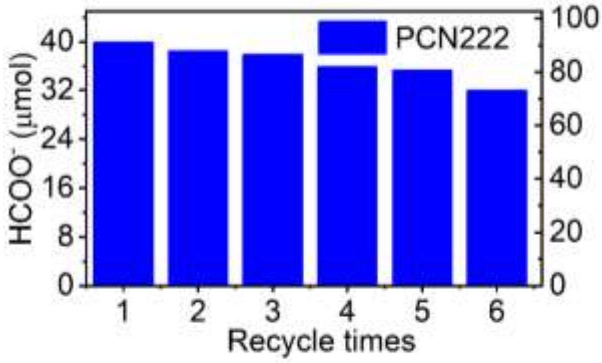

Figure S33. Generation of $HCOO^-$ in stability tests for PCN-222.

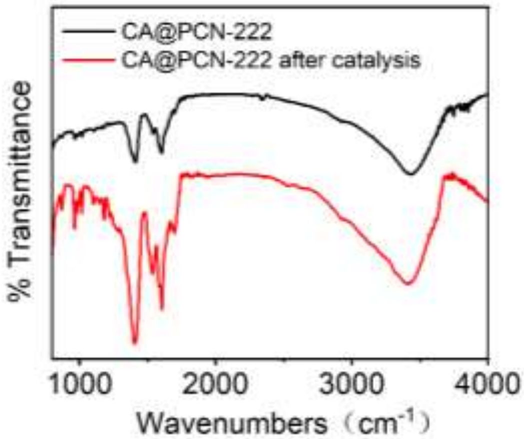

Figure S34. FT-IR for CA@PCN-222 before and after photocatalytic reaction, showing its well retained structure.

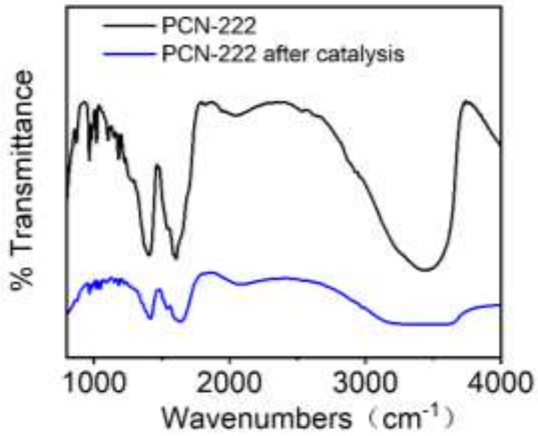

Figure S35. FT-IR for pristine PCN-222 before and after photocatalytic reaction.

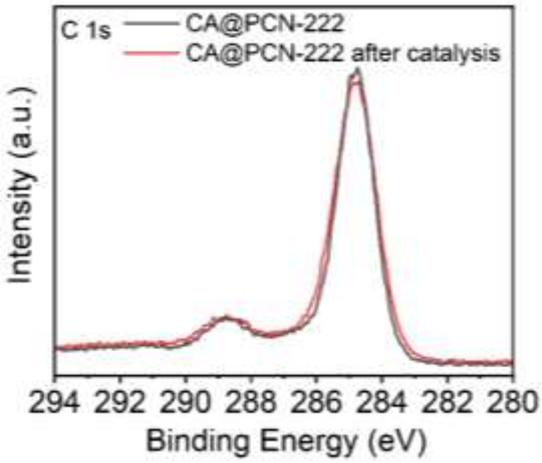

Figure S36. High–resolution XPS spectra of C 1s region for CA@PCN-222 before and after photocatalysis.

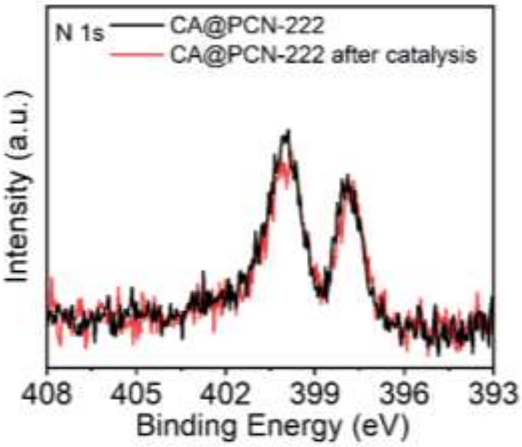

Figure S37. High-resolution XPS spectra of N 1s region for CA@PCN-222 before and after photocatalysis.

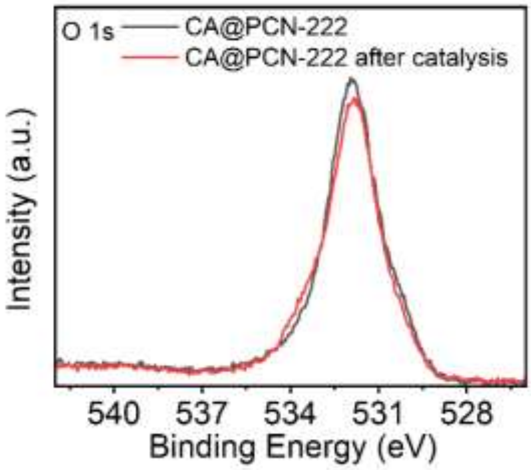

Figure S38. High-resolution XPS spectra of O 1s region for CA@PCN-222 before and after photocatalysis.

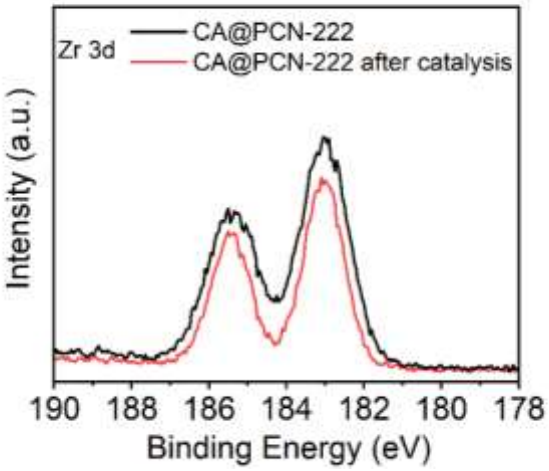

Figure S39. High-resolution XPS spectra of Zr 3d region for CA@PCN-222 before and after photocatalysis.

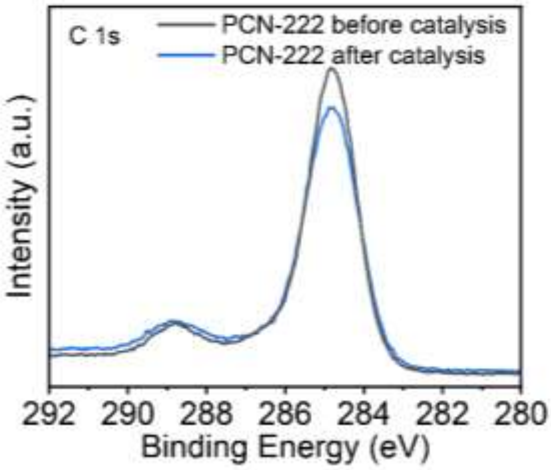

Figure S40. High-resolution XPS spectra of C 1s region for pristine PCN-222 before and after photocatalysis.

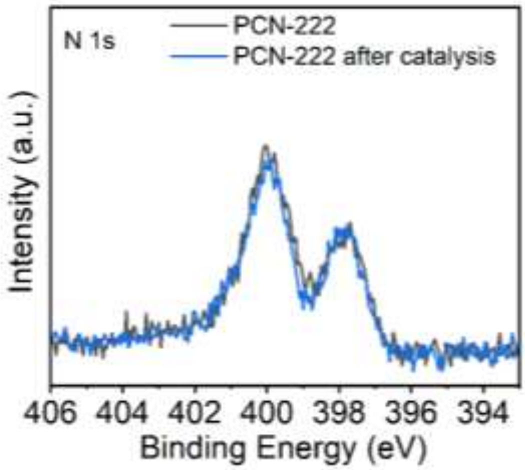

Figure S41. High-resolution XPS spectra of N 1s region for pristine PCN-222 before and after photocatalysis.

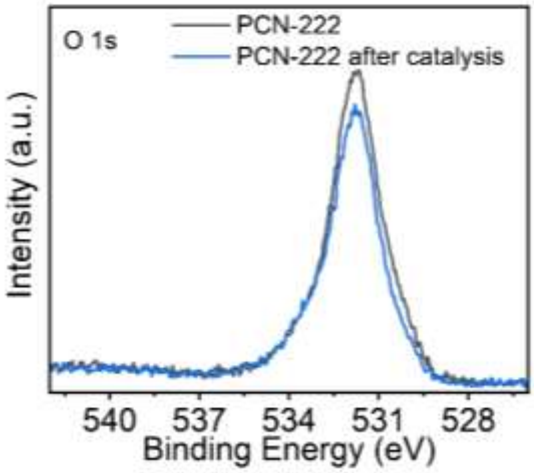

Figure S42. High-resolution XPS spectra of O 1s region for pristine PCN-222 before and after photocatalysis.

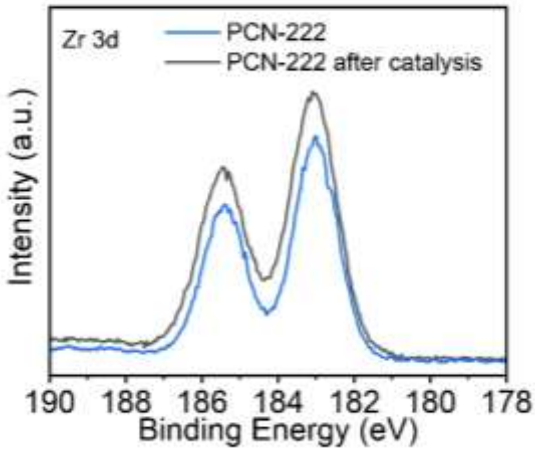

Figure S43. High-resolution XPS spectra of Zr 3d region for pristine PCN-222 before and after photocatalysis.

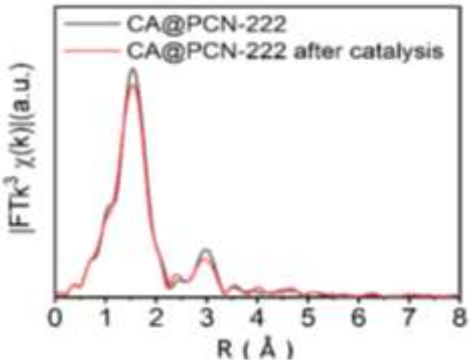

Figure S44. FT k^3-weighted Zr K-edge EXAFS spectra of CA@PCN-222 for before and after photocatalysis.

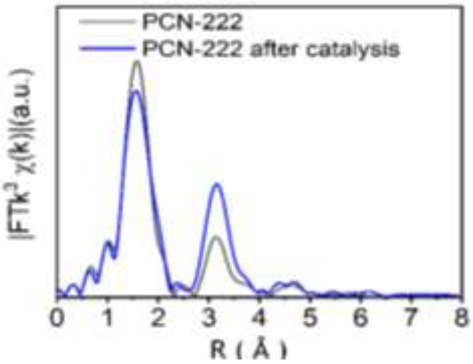

Figure S45. FT k^3-weighted Zr K-edge EXAFS spectra of CA@PCN-222 for before and after photocatalysis.

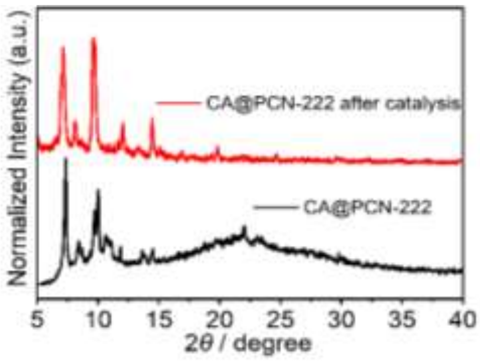

Figure S46. XRD of the CA@PCN-222 after photocatalytic reaction, showing its well retained structure.

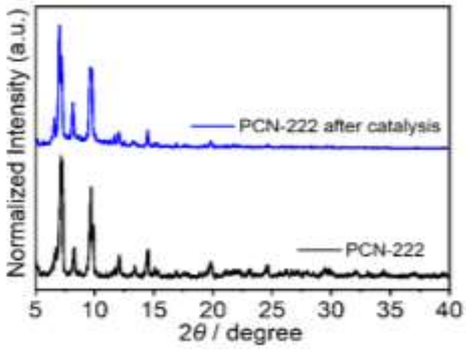

Figure S47. XRD of the PCN-222 after photocatalytic reaction, showing its well retained structure.

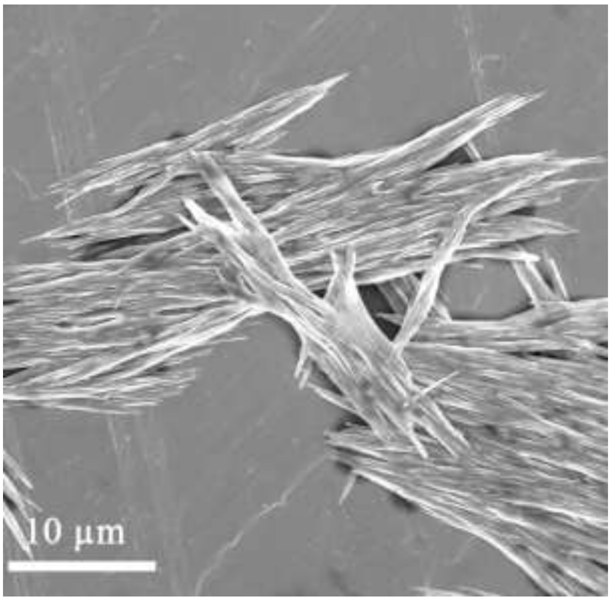

Figure S48. Sem after photocatalysis for CA@PCN-222.

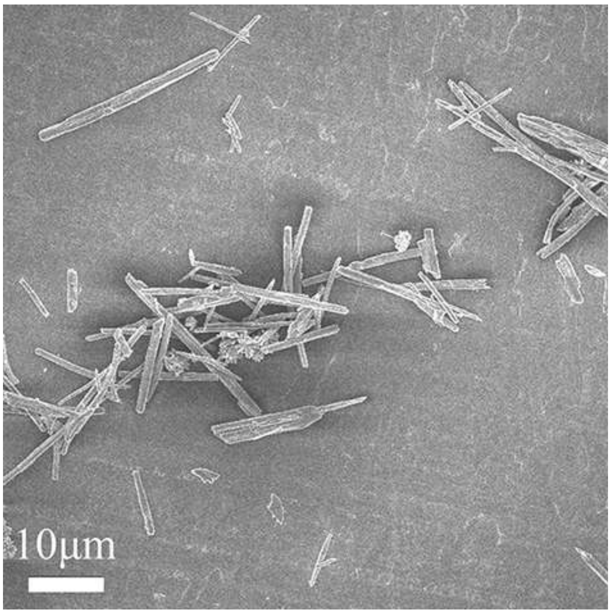

Figure S49. Sem after photocatalysis for pristine PCN-222.

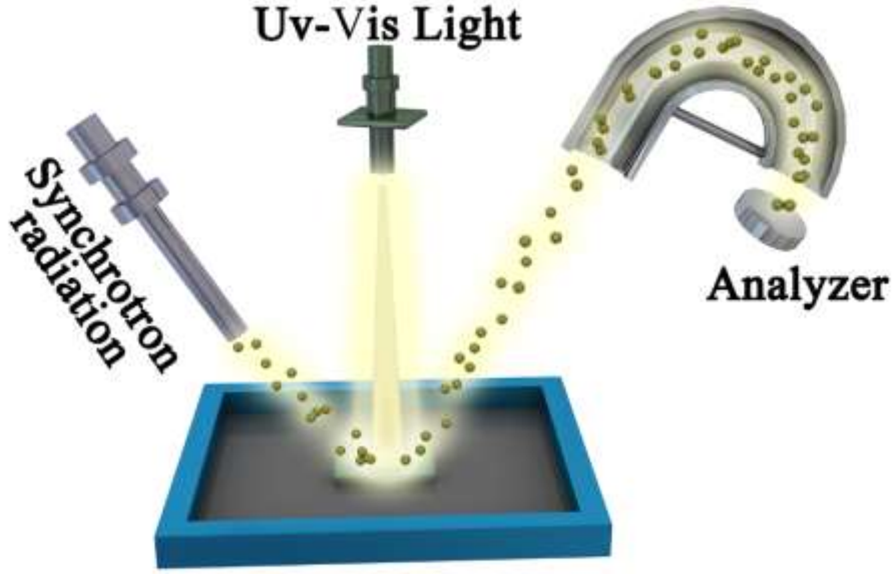

Figure S50. *In situ* XPS diagram for CO_2RR.

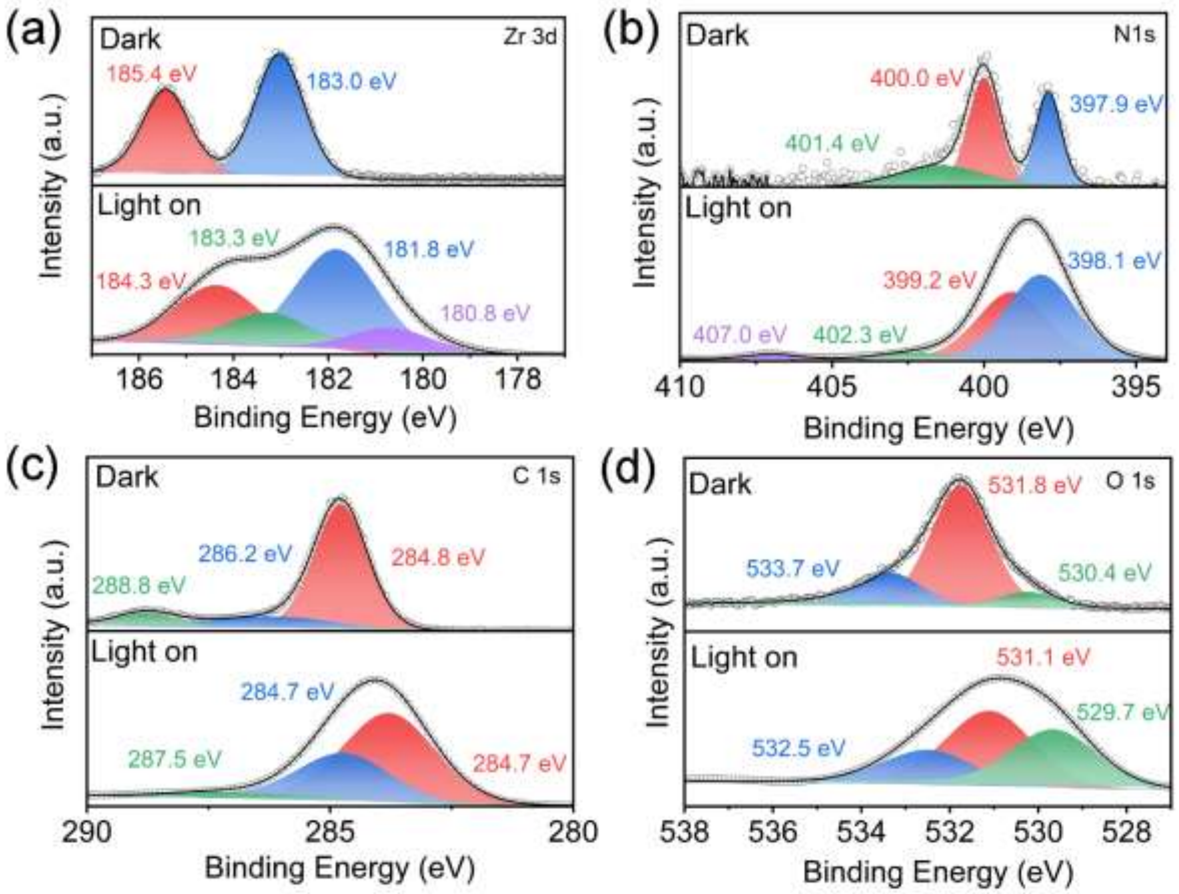

Figure S51. In situ high-resolution XPS spectra of CA@PCN-222 on irradiation at room temperature: (a) Zr 3d; (b) N 1s; (d) C 1s; (e) O 1s.

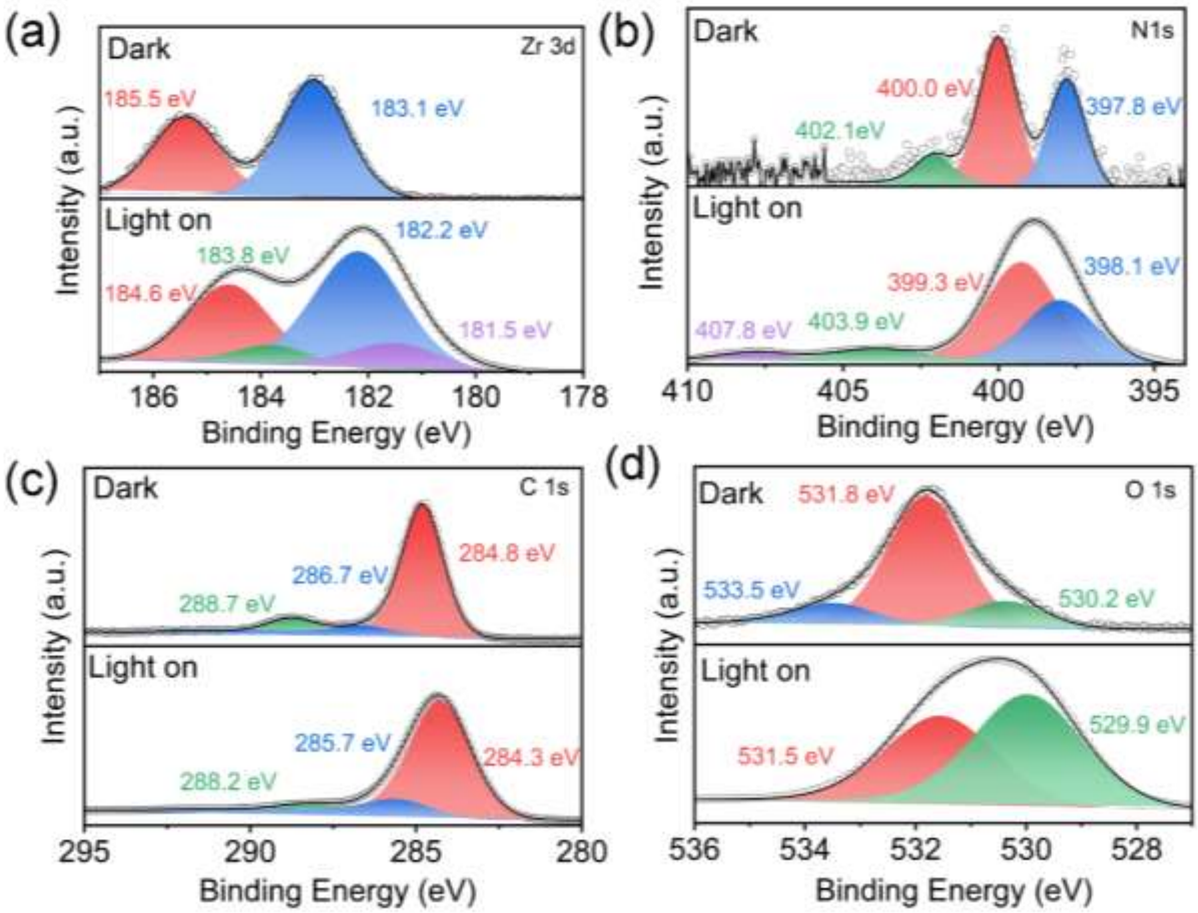

Figure S52. In situ high-resolution XPS spectra of PCN-222 on irradiation at room temperature: (a) Zr 3d; (b) N 1s; (d) C 1s; (e) O 1s.

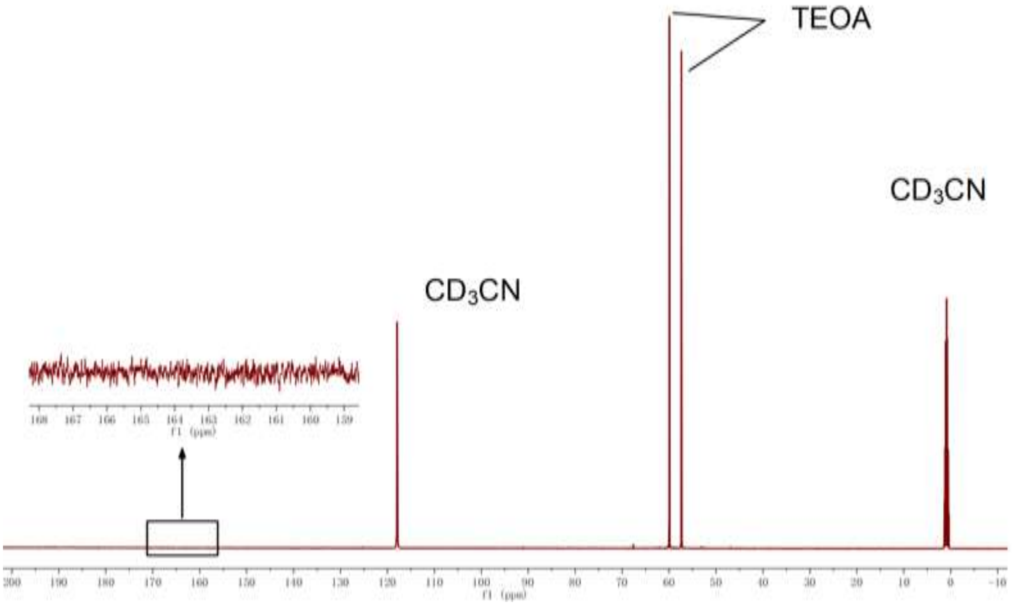

Figure S53. The ^{13}C NMR spectra for the product obtained from the reaction charging $^{12}CO_2$.

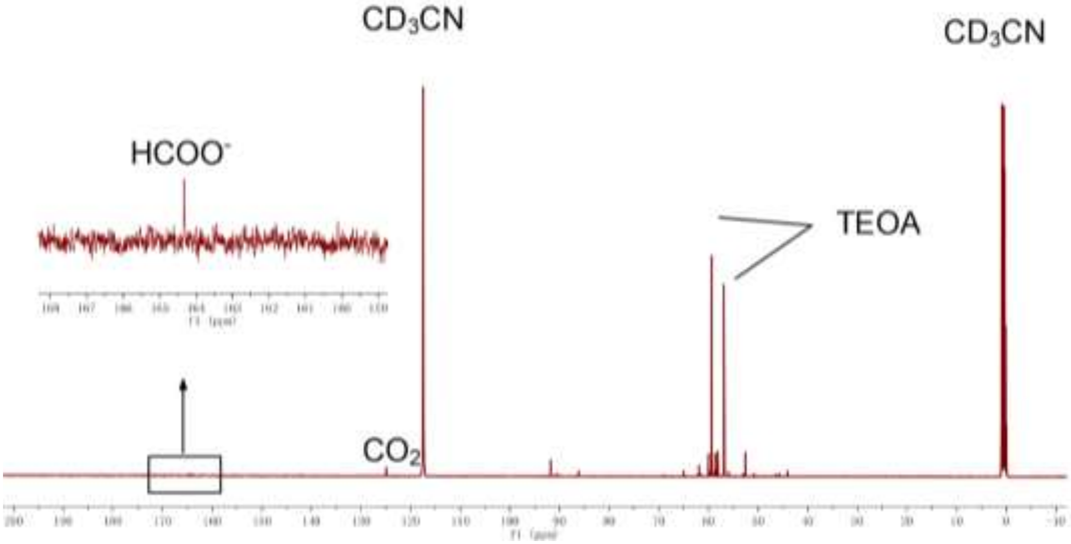

Figure S54. The ^{13}C NMR spectra for the product obtained from the reaction charging $^{13}CO_2$.

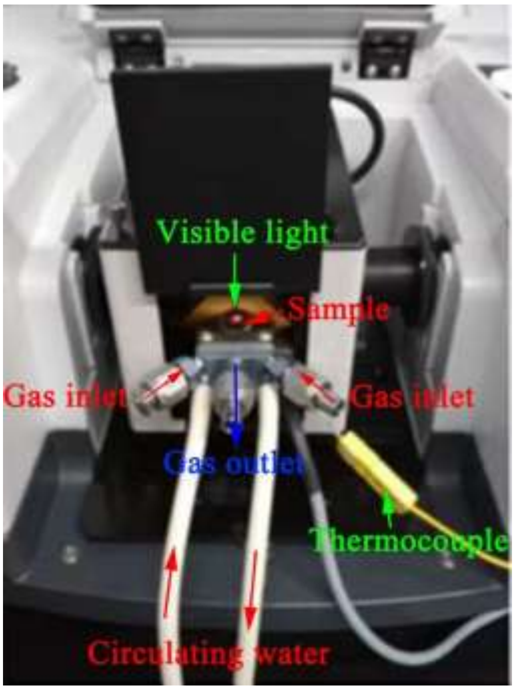

Figure S55. *In situ* infrared diagram for CO_2RR.

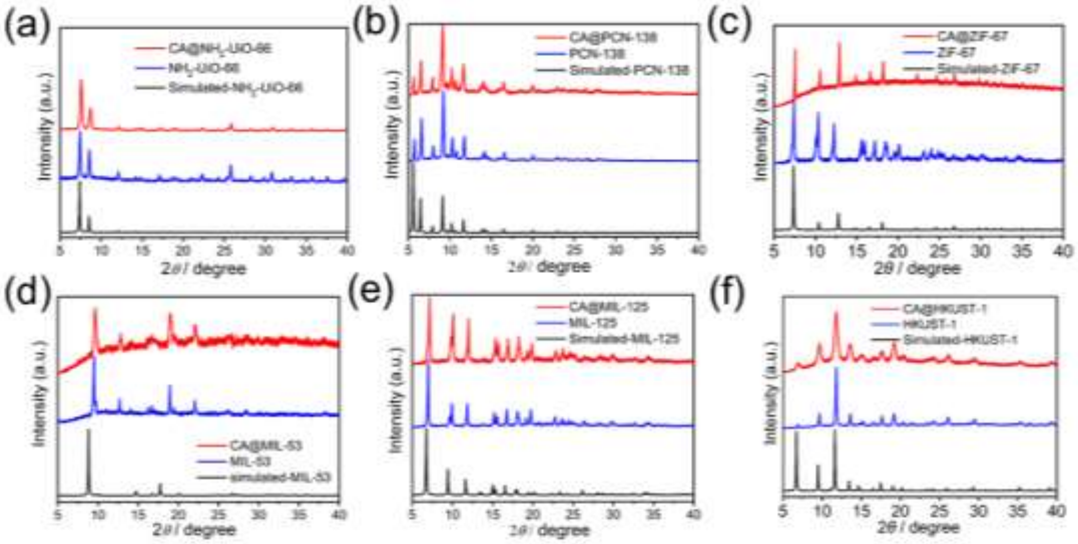

Figufre S56. The XRD of six kinds of MOFs as well as the MOF complexes with cellulose acetate.

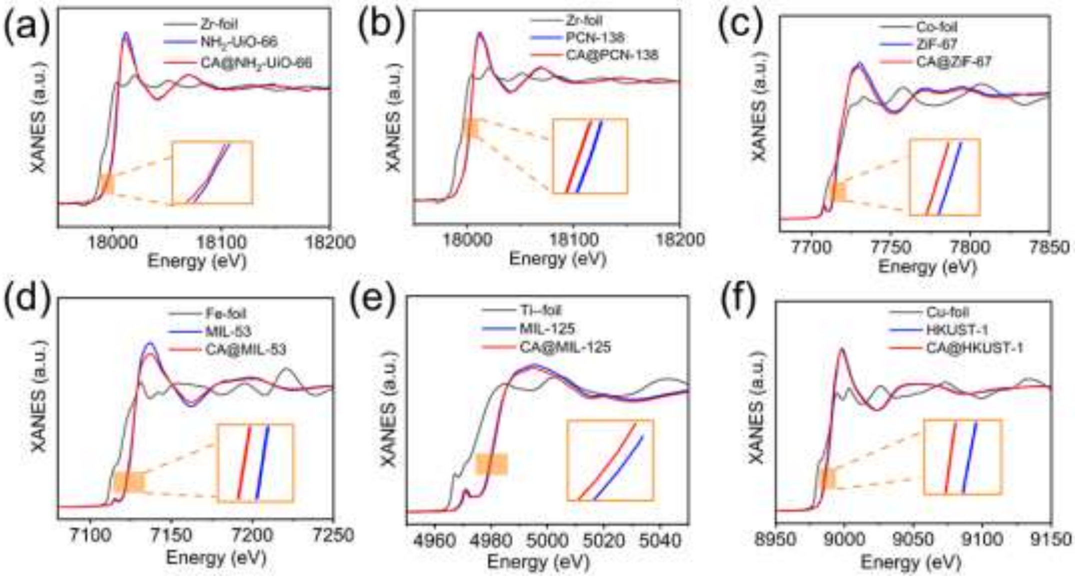

Figufre S57. The XANES of six kinds of MOFs as well as the MOF complexes with cellulose acetate.

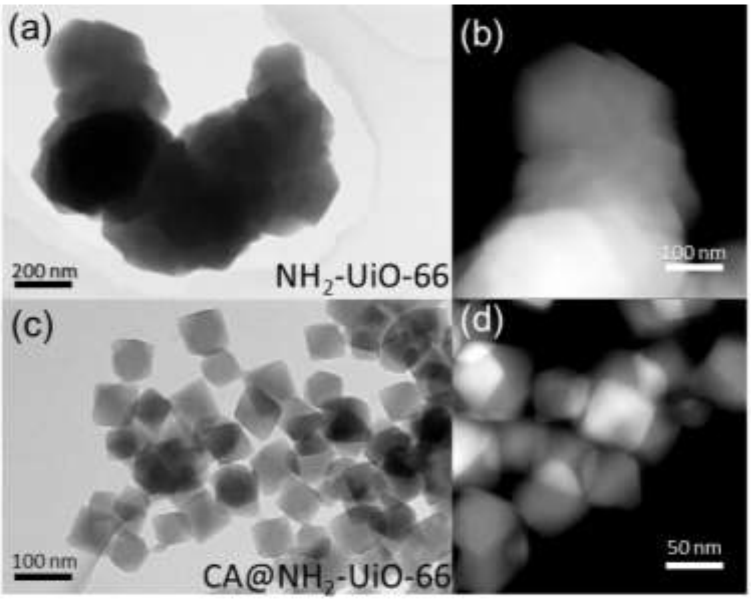

Figufre S58. (a)TEM image and (b) STEM image and EDX mapping of NH2-UiO-66; (c)TEM image and (d) STEM image and EDX mapping of CA@NH2-UiO-66.

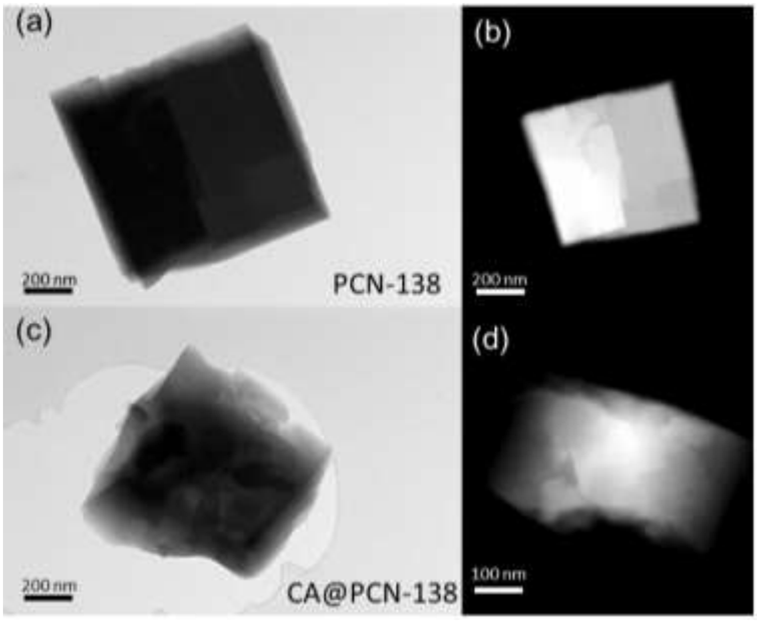

Figufre S59. (a)TEM image and (b) STEM image and EDX mapping of PCN-138; (c)TEM image and (d) STEM image and EDX mapping of CA@PCN-138.

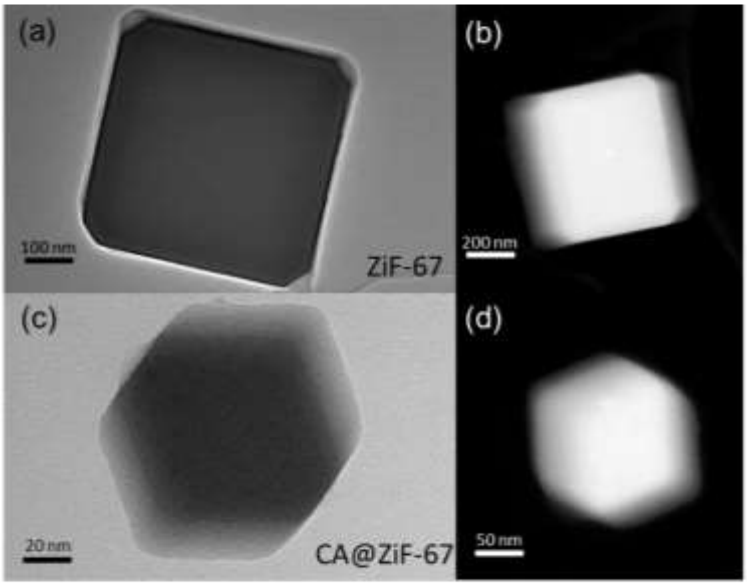

Figufre S60 (a)TEM image and (b) STEM image and EDX mapping of ZiF-67; (c)TEM image and (d) STEM image and EDX mapping of CA@ZiF-67.

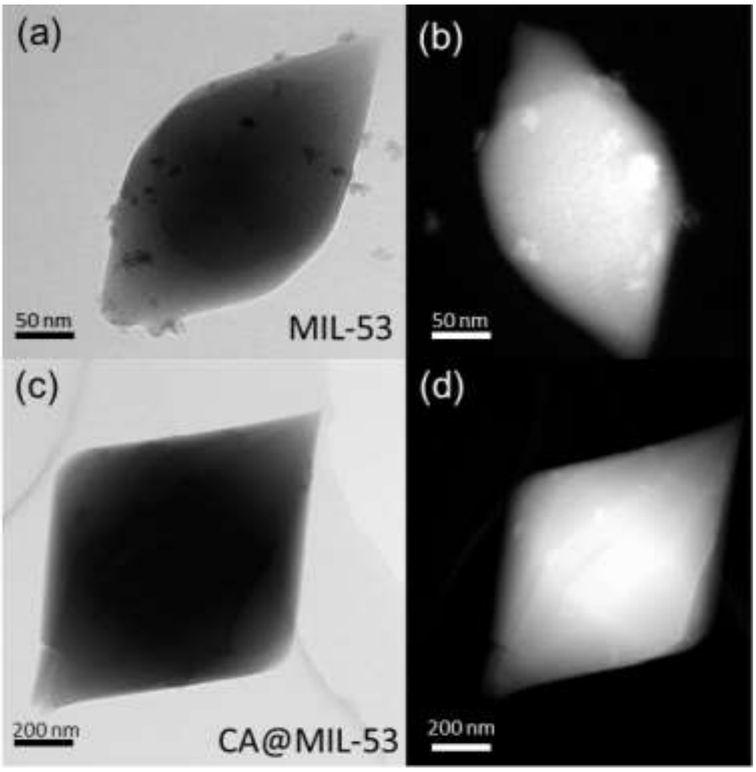

Figufre S61 (a)TEM image and (b) STEM image and EDX mapping of MIL53; (c)TEM image and (d) STEM image and EDX mapping of CA@MIL-53.

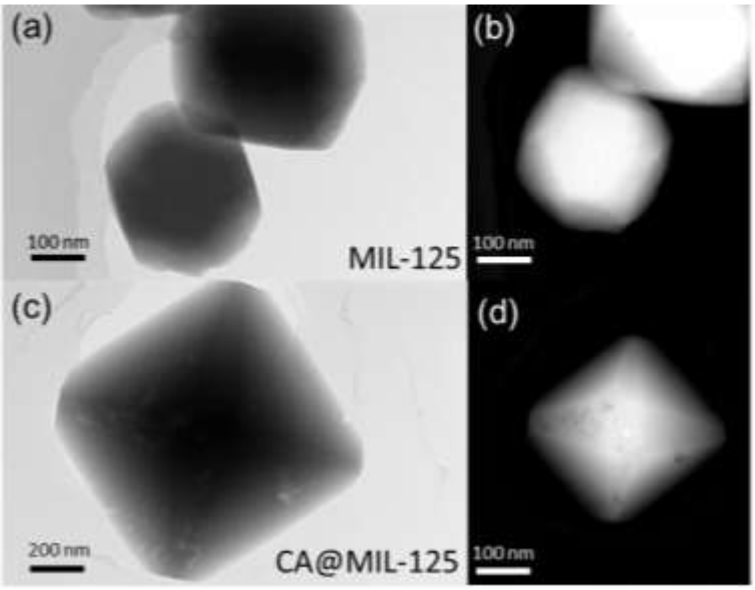

Figufre S62. (a)TEM image and (b) STEM image and EDX mapping of MIL-125; (c)TEM image and (d) STEM image and EDX mapping of CA@MIL-125.

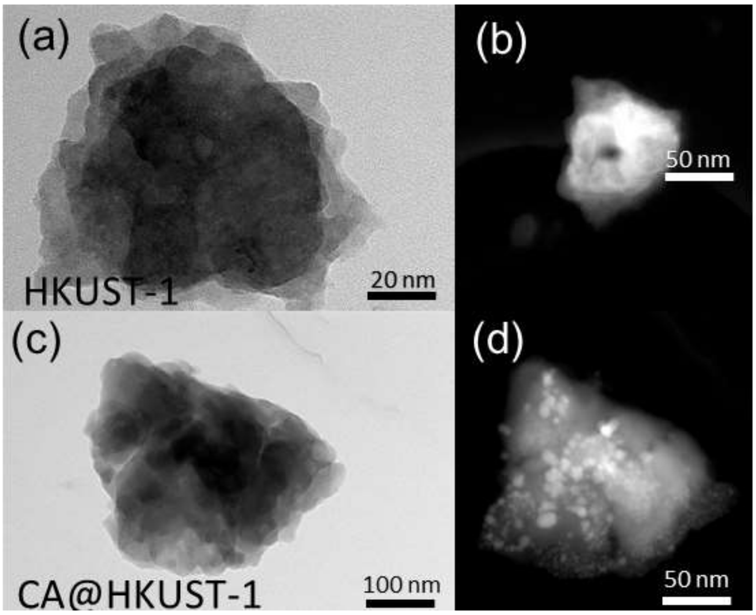

Figufre S63. (a)TEM image and (b) STEM image and EDX mapping of HKUST-1; (c)TEM image and (d) STEM image and EDX mapping of CA@HKUST-1.

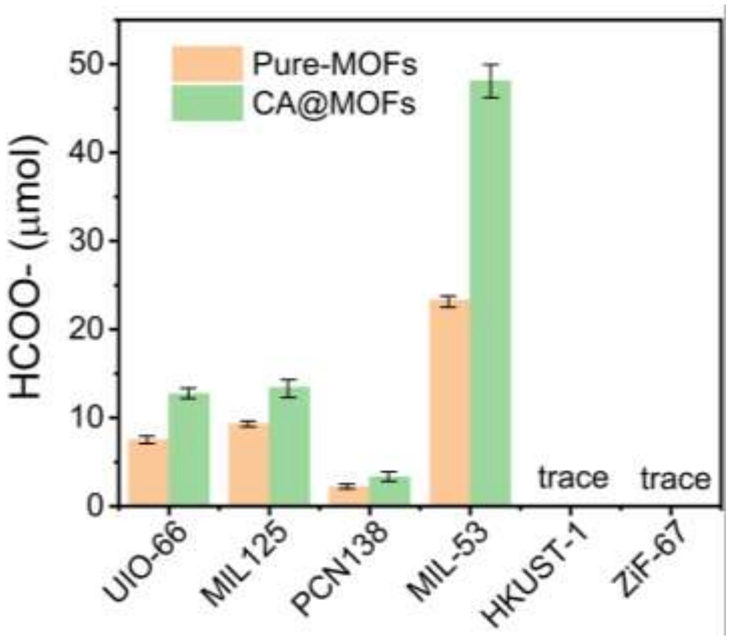

Figufre S64. UV/vis spectra of CA@PCN-222, PCN-222, H2TCPP, Zr-cluster and cellulose acetate (CA).

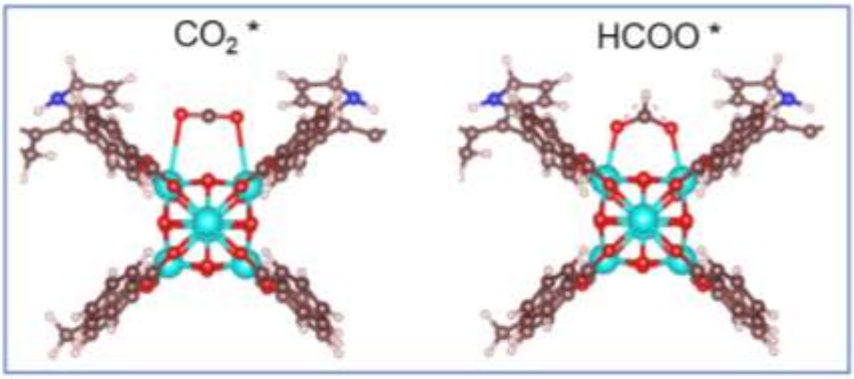

Figure S65. Diverse intermediates adsorbed onto an unsaturated Zr-oxo cluster in the catalytic CO_2RR process, for pristine PCN-222. Color scheme: Zr, cyan; C, brown; N, blue; O, red; H, white.

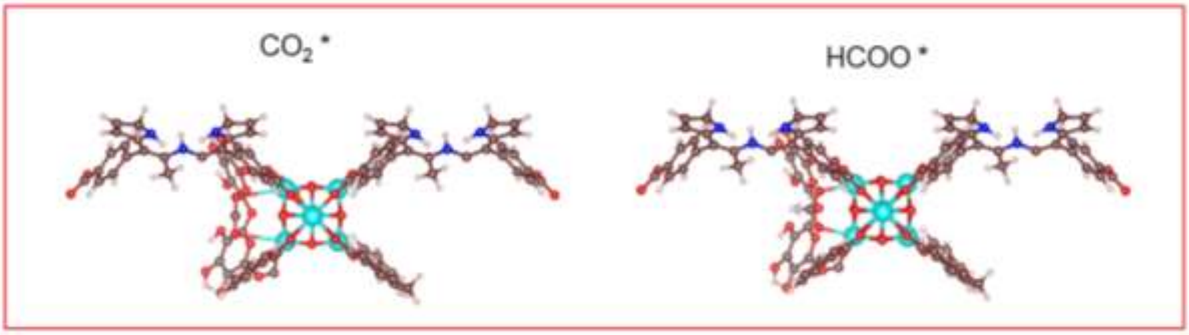

Figure S66. Diverse intermediates adsorbed onto an unsaturated Zr-oxo cluster in the catalytic CO_2RR process, for CA@PCN-222. Color scheme: Zr, cyan; C, brown; N, blue; O, red; H, white.

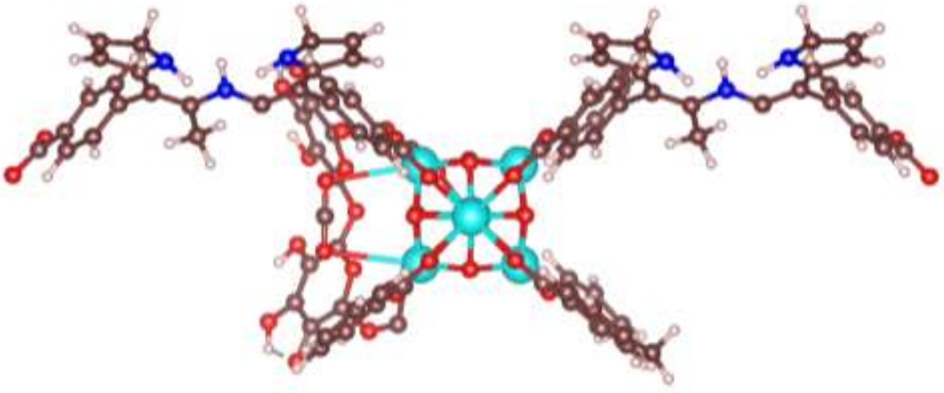

Figure S67. CO_2 adsorbed onto an unsaturated Zr–oxo cluster in the catalytic CO_2RR process for CA@PCN-222 (front view).

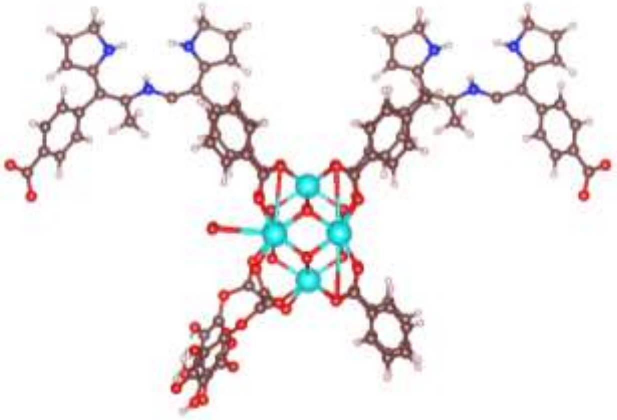

Figure S68. CO_2 adsorbed onto an unsaturated Zr–oxo cluster in the catalytic CO_2RR process for CA@PCN-222 (side view).

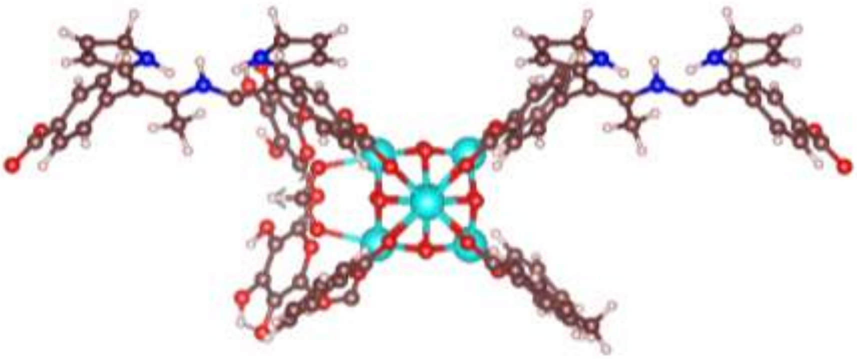

Figure S69. HCOO* adsorbed onto an unsaturated Zr–oxo cluster in the catalytic CO_2RR process for CA@PCN-222 (front view).

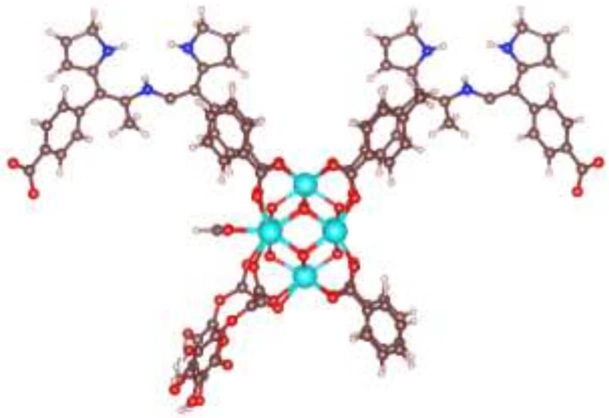

Figure S70. HCOO* adsorbed onto an unsaturated Zr–oxo cluster in the catalytic CO_2RR process for CA@PCN-222 (side view).

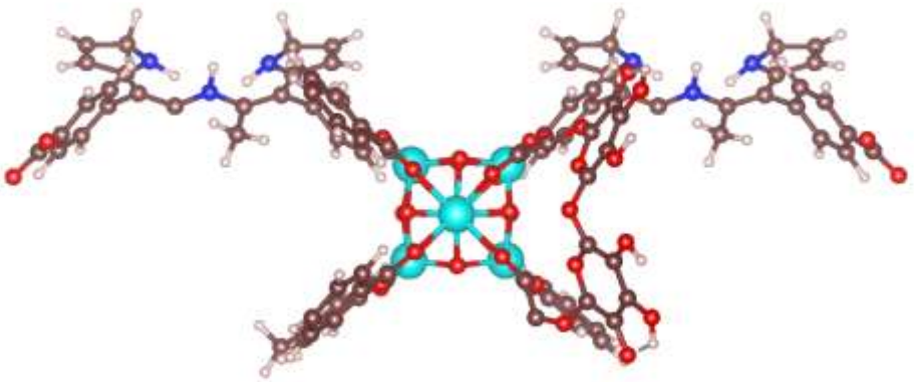

Figure S71. Slab* adsorbed onto an unsaturated Zr–oxo cluster in the catalytic CO_2RR process for CA@PCN-222 (front view).

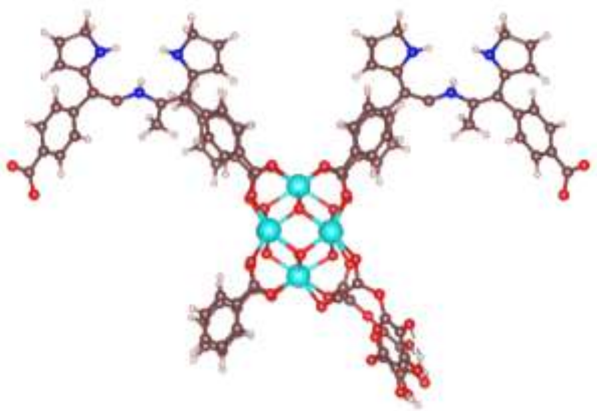

Figure S72 Slab* adsorbed onto an unsaturated Zr–oxo cluster in the catalytic CO_2RR process for CA@PCN-222 (side view).

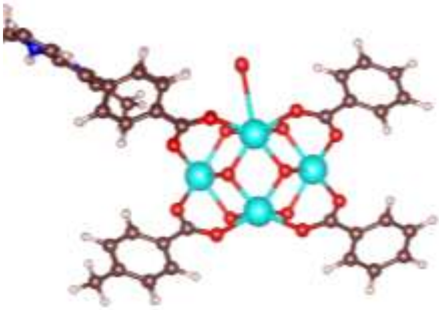

Figure S73. CO_2 adsorbed onto an unsaturated Zr–oxo cluster in the catalytic CO_2RR process for PCN-222 (front view).

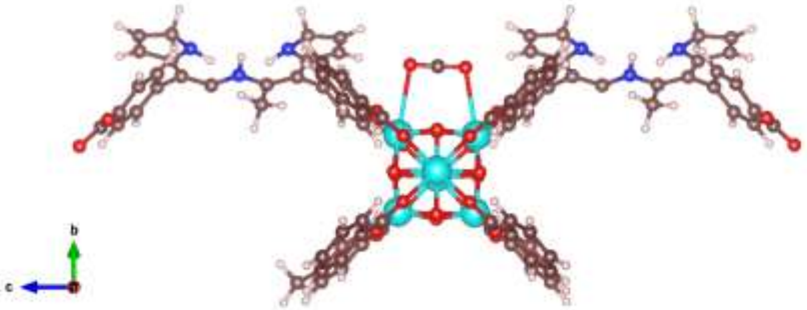

Figure S74. CO_2 adsorbed onto an unsaturated Zr–oxo cluster in the catalytic CO_2RR process for PCN-222 (side view).

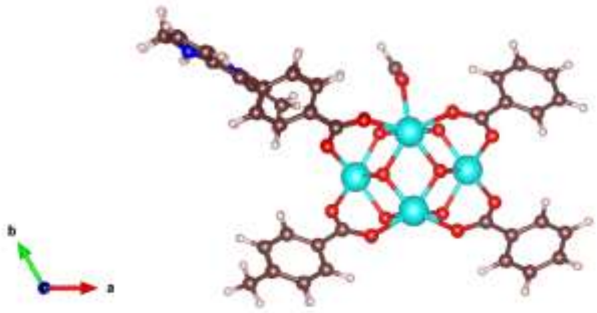

Figure S75. HCOO* adsorbed onto an unsaturated Zr–oxo cluster in the catalytic CO_2RR process for PCN-222 (front view).

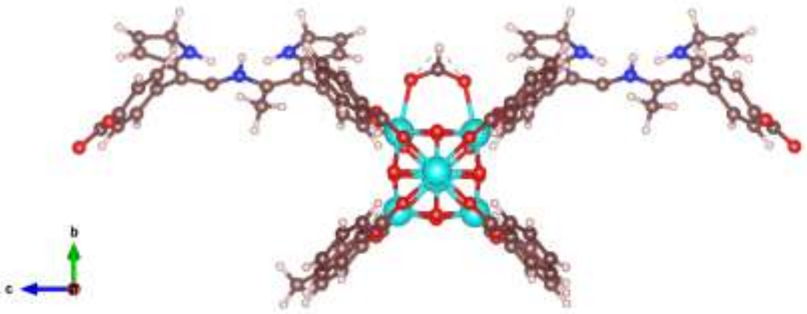

Figure S76. HCOO* adsorbed onto an unsaturated Zr–oxo cluster in the catalytic CO_2RR process for PCN-222 (side view).

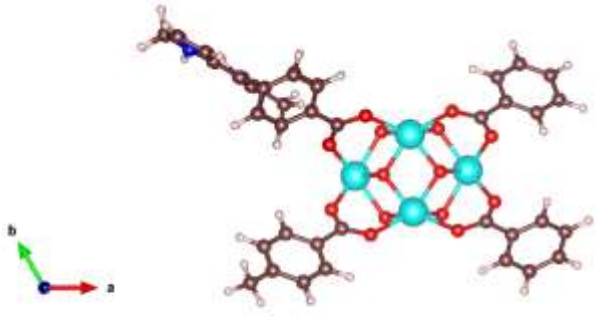

Figure S77. Slab* adsorbed onto an unsaturated Zr–oxo cluster in the catalytic CO_2RR process for PCN-222 (front view).

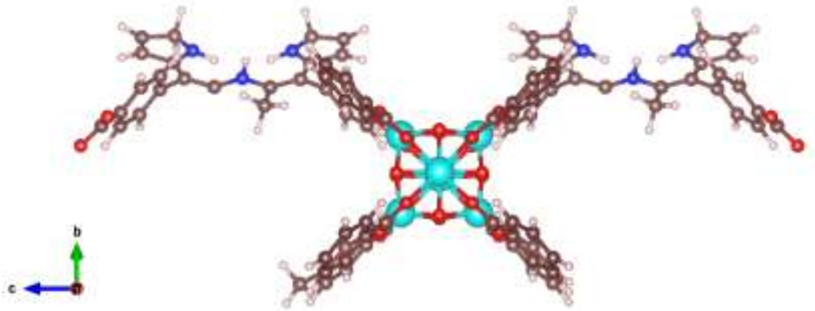

Figure S78. Slab* adsorbed onto an unsaturated Zr–oxo cluster in the catalytic CO_2RR process for PCN-222 (side view).

Reference

(1) Ling, L.L.; Yang, W.J.;Yan,; P.; Wang, M.; Jiang, H.L., Angew. Chem. Int. Ed. **2022**, 61(12): e202116396.
(2) Hu, M.L.; Liu, J.H.; Song, S.J.; Wang, W.W.; Yao, J.S.; Gong, Y.X.; Li, C.Y.; Li, H.; Li, Y.J.; Yuan, X.L.; Fang, Z.; Xu, H.; Song, YW.Y.; Li, Z.X., ACS Catalysis, **2022**, 12(5): 3238-3248.
(3) Wang, V.; Xu, N.; Liu, J.C.; Tang, G.; Geng, W.T., Computer Physics Communications, **2021**, 267: 108033.
(4) Xu, J.Q.; Ju, Z.Y.; Zhang, W.; Pan, Y.; Zhu, J.F.; Mao, J.W.; Zheng, L.X.; Fu, H.Y.; Yuan, M.L.; Chen, H.; Li, R.X., Angew. Chem. Int. Ed., **2021**, 60(16): 8705-8709.
(5) Zu, X.L.; Li, X.D.; Liu, W.; Sun, Y.F.; Xu,J.Q.; Yao, T.; Yan, W.S.; Gao, S.; Wang, C.M.; Wei, S.Q.; Xie, Y., Advanced Materials, **2019**, 31(15): 1808135.
(6) Xu, H. Q.; Hu, J.; Wang, D.; Li, Z.; Zhang, Q.; Luo, Y.; Yu, S. H.; Jiang, H. L., J. Am. Chem. Soc. **2015**, 137 (42), 13440-3. DOI: 10.1021/jacs.5b08773
(7) Fu, Y.; Sun, D.; Chen, Y.; Huang, R.; Ding, Z.; Fu, X.; Li, Z., Angew. Chem. Int. Ed. **2012**, 51, 3364-3367.
(8) Sun, D.; Gao, Y.; Fu, J.; Zeng, X.; Chen, Z.; Li, Z., Chem. Commun. **2015**, 51, 2645-2648.
(9) Sun, D.; Liu, W.; Qiu, M.; Zhang, Y.; Li, Z., Chem. Commun. **2015**, 51, 2056-2059.
(10) Chen, D.; Xing, H.; Wang, C.; Su, Z., J. Mater. Chem. A **2016**, 4, 2657-2662.
(11) Wang, D.; Huang, R.; Liu, W.; Sun, D.; Li, Z., ACS Catal. **2014**, 4, 4254-4260.
(12) Fu, Y.; Sun, D.; Chen, Y.; Huang, R.; Ding, Z.; Fu, X.; Li, Z., Angew. Chem. Int. Ed. **2012**, 51, 3364-3367.
(13) Lee, Y.; Kim, S.; Kang, J. K.; Cohen, S. M., Chem. Commun. **2015**, 51, 5735-5738.
(14) Li, X.-X.; Liu, J.; Zhang, L.; Dong, L.-Z.; Xin, Z.-F.; Li, S.-L.; Huang-Fu, X.-Q.; Huang, K.; Lan, Y.-Q., ACS Appl. Mater. Interfaces **2019**, 11, 25790-25795.
(15) Dong, L.-Z.; Zhang, L.; Liu, J.; Huang, Q.; Lu, M.; Ji, W.-X.; Lan, Y.-Q., Angew. Chem. Int. Ed. **2020**, 59, 2659-2663.
(16) Zhang, S.; Li, L.; Zhao, S.; Sun, Z.; Luo, J., Inorg. Chem. **2015**, 54, 8375-8379.
